# Extra-coding RNAs regulate neuronal DNA methylation dynamics

**DOI:** 10.1038/ncomms12091

**Published:** 2016-07-07

**Authors:** Katherine E. Savell, Nancy V. N. Gallus, Rhiana C. Simon, Jordan A. Brown, Jasmin S. Revanna, Mary Katherine Osborn, Esther Y. Song, John J. O'Malley, Christian T. Stackhouse, Allison Norvil, Humaira Gowher, J. David Sweatt, Jeremy J. Day

**Affiliations:** 1 Department of Neurobiology, McKnight Brain Institute, University of Alabama at Birmingham, Birmingham, Alabama 35294, USA; 2 Department of Biochemistry, Purdue University, West Lafayette, Indiana 47907, USA

## Abstract

Epigenetic mechanisms such as DNA methylation are essential regulators of the function and information storage capacity of neurons. DNA methylation is highly dynamic in the developing and adult brain, and is actively regulated by neuronal activity and behavioural experiences. However, it is presently unclear how methylation status at individual genes is targeted for modification. Here, we report that extra-coding RNAs (ecRNAs) interact with DNA methyltransferases and regulate neuronal DNA methylation. Expression of ecRNA species is associated with gene promoter hypomethylation, is altered by neuronal activity, and is overrepresented at genes involved in neuronal function. Knockdown of the *Fos* ecRNA locus results in gene hypermethylation and mRNA silencing, and hippocampal expression of *Fos* ecRNA is required for long-term fear memory formation in rats. These results suggest that ecRNAs are fundamental regulators of DNA methylation patterns in neuronal systems, and reveal a promising avenue for therapeutic targeting in neuropsychiatric disease states.

## Abstract (web summary)

DNA methylation in the brain is a dynamic process, but gene-specific regulation of this process is poorly understood. Here, Day and colleagues show that extra-coding RNAs interact with DNA methyltransferases and regulate neuronal DNA methylation to control gene expression in locus-specific manner in neurons.

## Graphical abstract

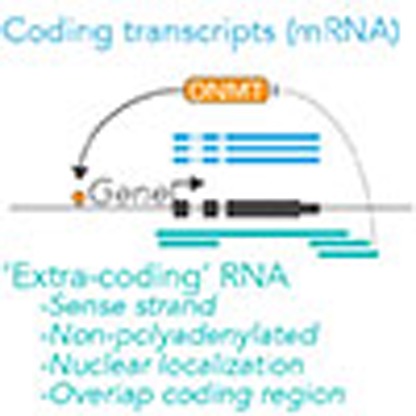

Methylation of cytosine bases in DNA is a critical regulator of the function and plasticity within the central nervous system. In the developing brain, neurons exhibit unique DNA methylation patterns that are correlated with synaptogenesis and regulate the expression of neuronal genes^1^. In the adult brain, active DNA methylation is required for memory formation and maintenance^2^,^3^,^4^,^5^, and neuronal activity and behavioural experiences lead to site-specific reorganization of DNA methylation dynamics^4^,^6^,^7^,^8^,^9^. In addition, maintenance of DNA methylation patterns at specific genes is disrupted in numerous cognitive and neurodegenerative disorders^10^,^11^,^12^,^13^,^14^.

Despite the well-appreciated role of DNA methylation in neuronal function and physiology, the mechanisms by which individual genes or sequences of DNA are targeted for active methylation or demethylation are presently unclear. Emerging evidence from other systems suggests that site-specific regulation of DNA methylation can occur via non-coding RNA species^15^,^16^,^17^,^18^. Most remarkably, extra-coding RNAs (ecRNAs), previously characterized as non-polyadenylated, sense-strand RNA overlapping protein-coding genes, have been shown to bind to DNA methyltransferases (DNMTs) and direct gene-specific methylation patterns^15^. These RNAs were previously found to precede synthesis of its messenger RNA (mRNA) counterpart in S phase and to be regulated by both RNA polymerases II and III (ref. 15). However, the extent and nature of this regulation in post-mitotic cells such as neurons has not been explored.

Here, we report that ecRNA species, which we define as sense-strand, non-polyadenylated RNAs that overlap with the gene boundaries, are generated from genes that are critical for neuronal responses to stimulation, and that neuronal activity modifies ecRNA production. Genome-wide, ecRNA levels are correlated with mRNA expression and promoter DNA methylation. We show that ecRNAs directly bind DNA methyltransferases to inhibit their activity, and that blockade of ecRNA production at the *Fos* gene locus results in hypermethylation and gene silencing. Finally, our data reveal that ecRNA expression from the *Fos* gene in the hippocampus is required for the formation of long-term fear memories, suggesting a critical role for ecRNA-directed DNA methylation in neuronal function within the adult brain.

## Results

### Genome-wide classification of neuronal ecRNAs

**Figure 1:**
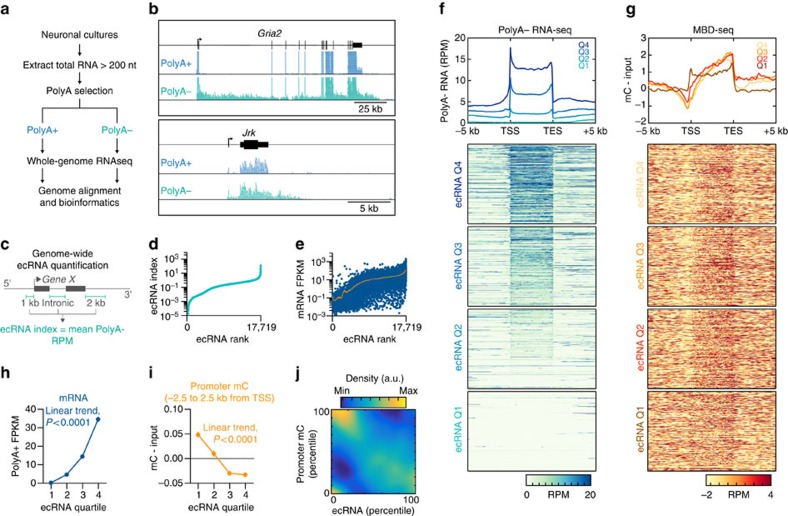
Genome-wide identification and quantification of ecRNAs from neuronal systems. (**a**) RNA-seq workflow identifies both polyadenylated and non-polyadenylated transcripts from the same neuronal tissue. (**b**) Comparison of PolyA+ and PolyA− sequencing from representative gene loci reveals PolyA− transcripts arising from intronic and post-TESs. (**c**) Genome wide, extra-coding transcripts were characterized by averaging PolyA− reads that mapped to 5′ (pre-TSS), intronic or 3′ (post-TES) of a given gene. (**d**) Rank plot of ecRNA index at 17,719 rat genes. (**e**) mRNA expression (PolyA+ RNA-seq) ranked by ecRNA index reveals correlation between ecRNA and mRNA expression. (**f**) Division of ecRNA into discrete quartiles reveals general profile and expression of PolyA− RNA transcripts. Data are aligned to transcription start sites (TSS) and TESs. Heatmap shows PolyA− transcription from all genes. (**g**) MBD-seq reveals metagenomic DNA methylation profiles, including hypomethylation at TSS and hypermethylation at TES. ecRNA transcription is associated with hypomethylated promoters across the genome. (**h**,**i**) Genome wide, ecRNA levels are positively correlated with mRNA transcription ((**h**) one-way ANOVA, F_(3,17715)_=612.5, *P*<0.0001) and negatively correlated with promoter DNA methylation ((**i**) one-way ANOVA, F_(3,17715)_=73.27, *P*<0.0001). (**j**) Percentile-percentile density scatterplot of ecRNA and promoter DNA methylation. Data in **h** and **i** are presented as mean±s.e.m.

To characterize the prevalence and expression of ecRNAs in neuronal systems, we established a pipeline for detection of non-polyadenylated (PolyA−) RNA transcripts using whole-genome, directional RNA sequencing (Fig. 1a). Total RNA was extracted from neuronal cultures prepared from embryonic rat cortex, and underwent selection for polyadenylation signals. PolyA+ and PolyA− libraries underwent identical sequencing and were aligned to the rat genome (Rn5 build) using Tophat. This pipeline resulted in considerable enrichment of known non-polyadenylated transcripts (for example, genes encoding canonical histone proteins) in the PolyA− sequencing tracks, as well as highly reproducible PolyA+ and PolyA− sequencing between biological replicates (Supplementary Fig. 1). Comparison between PolyA+ and PolyA− RNA-seq data sets revealed the presence of non-polyadenylated, ecRNA-like transcripts overlapping protein-coding genes that also generate PolyA+ transcripts (Fig. 1b). Although a considerable fraction of PolyA− transcripts mapped to exonic regions (possibly indicative of spliced mRNA that had not yet been polyadenylated), we also observed frequent read coverage from intronic regions and regions outside the typical gene boundaries (transcription start and end sites) specific to the PolyA− library (Fig. 1b, Supplementary Fig. 1c). To characterize the genome-wide expression of ecRNA-like transcripts, we computed an ecRNA index of sense-strand PolyA− reads arising from upstream, intronic and downstream loci surrounding genes (Fig. 1c). This index revealed that ecRNA-like expression from known coding genes was ubiquitous across the genome, and was highly correlated with mRNA expression from the overlapping gene (Fig. 1d,e).

To determine whether ecRNA production was associated with DNA methylation states genome-wide, we next performed whole-genome sequencing on methylated DNA fragments enriched with a recombinant methyl-binding domain (MBD) protein (Supplementary Fig. 2). As demonstrated previously^19^, we observed hypomethylation of the vast majority of gene promoters and CpG islands within the genome, and hypermethylation of gene bodies across the entire genome (Fig. 1g, Supplementary Fig. 3a,b). To examine whether ecRNA levels reflected DNA methylation patterns, we divided all genes into distinct quartiles according to their ecRNA index (Fig. 1f–g). This analysis revealed genes with higher ecRNA expression generated significantly more mRNA and possessed less DNA methylation at promoters (defined as 5 kb region surrounding the transcription start site (TSS); Fig. 1h–j). As this effect could potentially be driven by the presence of CpG islands at highly expressed genes, we next examined the relationship between ecRNA levels and CpG island status. As expected, promoters containing CpG islands were hypomethylated, whereas promoters lacking CpG islands were hypermethylated (Supplementary Fig. 3c). However, the relationship between ecRNA level and promoter DNA methylation was preserved regardless of promoter CpG status. Additionally, we found that DNA methylation at gene-overlapping CpG islands was strongly related to ecRNA expression (Supplementary Fig. 3d), indicative of potential ecRNA regulation of DNA methylation states at both promoter and intragenic sites.

**Figure 2:**
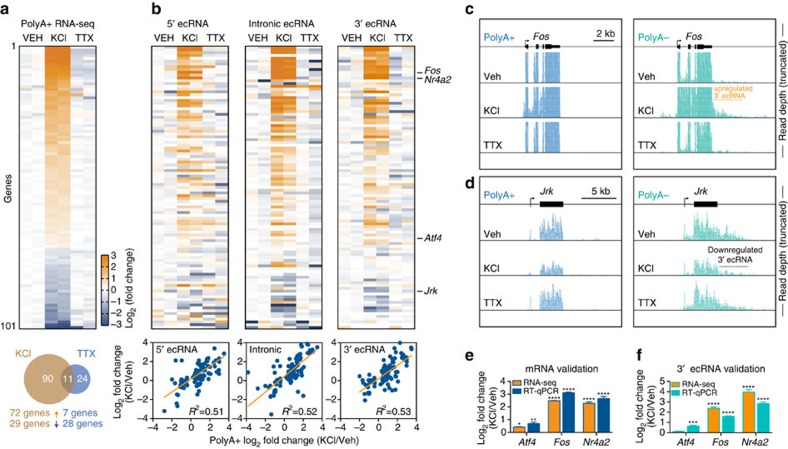
Regulation of mRNA and ecRNA by neuronal activity. (**a**) PolyA+ RNA-seq following 1 h neuronal depolarization (25 mM KCl) or inactivation (1 μM TTX) reveals altered mRNA expression at a small subset of genes. Top, heatmap of KCl-altered transcripts (each column=1 biological replicate; 2 replicates per treatment). Bottom, Venn diagram of overlap between transcripts altered by KCl and TTX. (**b**) Corresponding heatmaps from PolyA− RNA-seq reveal relationship between activity-related mRNA and ecRNA changes. PolyA− RNA transcription from 5′, intronic and 3′ sites all correlated significantly with mRNA changes following neuronal depolarization with KCl (linear regression, *P*<0.0001 for each comparison). (**c**,**d**) Representative examples of activity-induced increases (*Fos* gene, (**c**)) and decreases (*Jrk* gene, (**d**)) in 3′ ecRNA levels. RNA-seq reads shown individually; read depth truncated to highlight ecRNA changes. (**e**,**f**) Validation of RNA-seq results with RT-qPCR (*n*=6 biological replicates) confirms changes in mRNA and ecRNA levels. Data are expressed as mean±s.e.m. Individual comparisons made with Student's *t*-test versus vehicle, *P<0.05, **P<0.01, ***P<0.001 and ****P<0.0001.

Given that DNA methylation status in neurons is both activity and experience-dependent, we next assessed the degree to which ecRNAs were altered by neuronal activity. Neurons were incubated with potassium chloride (KCl) or tetrodotoxin (TTX) (Fig. 2), which increase and decrease neuronal activity, respectively. We again performed genome-wide RNA sequencing to examine both PolyA+ (mRNA) and PolyA− (including extra-coding) RNA species. Following neuronal depolarization with KCl, we observed that PolyA+ expression from a small set of genes (Fig. 2a; Supplementary Data 1) were modified by neuronal activity, including a number of immediate early genes (*Fos*, *Arc*, *Egr1* and *Nr4a2*) that have well-established roles in neuronal and behavioural function. To determine whether non-polyadenylated transcripts arising from these activity-regulated genes were also modified by neuronal depolarization, PolyA− transcripts arising from the 5′, intronic, and 3′ extra-genic regions were plotted and compared with PolyA+ transcripts (Fig. 2b; Supplementary Data 1). Depolarization-induced ecRNA transcription from each of these regions was significantly correlated with PolyA+ transcription, suggesting that ecRNAs arising from these genes are also activity-dependent (see Fig. 2c,d for representative examples). Several of these results were independently verified using quantitative PCR with reverse transcription (RT-qPCR) with custom mRNA or ecRNA-specific primers (Fig. 2e,f), confirming significant activity-dependent regulation of ecRNA transcripts.

**Figure 3:**
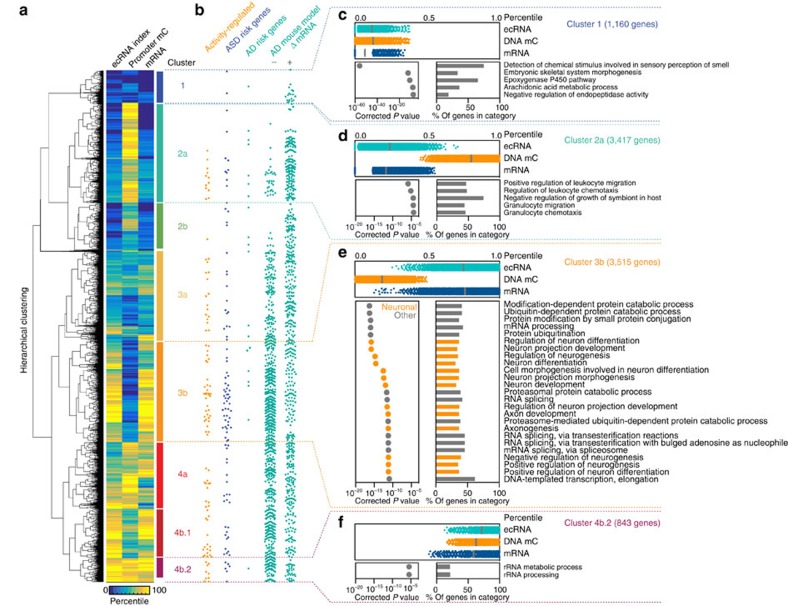
High ecRNA expression is inversely related to promoter DNA methylation at genes involved in neuronal function and brain disease. (**a**) Hierarchical clustering of percentile-normalized ecRNA, DNA mC and mRNA highlights major gene clusters. Clustering was performed using percentile scores instead of raw data to equalize data range. (**b**) Overlap between distinct gene clusters and activity-responsive (Veh versus KCl genes from Fig. 2a), genes harbouring mutations associated with autism spectrum-disorders^43^ (ASD risk genes), genes implicated in Alzheimer's disease risk^44^ (AD risk genes) and genes altered in an animal model of Alzhiemer's disease^34^ (AD mouse model ΔmRNA genes; downregulated (−) and upregulated (+)). Each circle represents a single gene. (**c**–**f**) Distribution of ecRNA, DNA mC and mRNA scores (top panels) and gene ontology analysis (bottom panels) of selected clusters. Gene ontology groups are ranked by corrected *P* value. Cluster 3b (**e**) is enriched for genes involved in neurogenesis, neuronal projection and neuronal development. This cluster represents 19.8% of genes investigated but contains 34% of activity-regulated genes (odds ratio=1.69; *P*=0.0079), 40% of the genes implicated in ASD (odds ratio=2.006; *P*=0.0003) and 39% of genes that are downregulated in a mouse AD model (odds ratio=1.966; *P*<0.0001).

To examine the interplay between ecRNA levels and DNA methylation status at different functional groups within the genome, we performed unbiased hierarchical clustering using a percentile-normalized quantification of ecRNA index, promoter DNA methylation and gene expression (mRNA fragments per kilobase of exon per million mapped reads (FPKM)). Hierarchical clustering identified at least eight distinct subgroups of genes (Fig. 3). As highlighted above, gene clusters with the highest ecRNA levels tended to have the lowest promoter methylation, whereas clusters that did not generate ecRNAs were characterized by promoter hypermethylation. Gene ontology analysis of the largest cluster demonstrated an enrichment of genes involved in neuronal processes, including gene ontology terms for neuronal differentiation, neuronal development and dendritic spine morphogenesis (Fig. 3e, Supplementary Data 2). This subclass of genes was marked by a high ecRNA index and low promoter DNA methylation, and also overlapped with genes regulated by neuronal depolarization and genes involved in intellectual/learning disorders and neurodegenerative disease states (Fig. 3b). In contrast, the second-largest cluster was marked by low ecRNA levels and high promoter DNA methylation, and was enriched for gene ontology-terms related to immune function (Fig. 3d).

Although the vast majority of genes followed the general inverse relationship between ecRNA levels and DNA methylation, one small cluster of genes was marked by promoter hypomethylation despite the relative absence of ecRNA expression. This category generated the lowest mRNA levels, and was heavily enriched with genes that code for olfactory receptors (Fig. 3c), which possess unique regulatory mechanisms that help to specify expression of a single olfactory receptor gene in olfactory sensory neurons^20^. Similarly, another small cluster was marked by promoter hypermethylation despite high ecRNA quantification and high mRNA expression (Fig. 3f). This cluster was enriched for genes involved in ribosomal RNA processing, which involves robust expression of genes that are critical for function in all cells. Overall, this analysis suggests that ecRNAs may be important for regulation of DNA methylation states at certain functional classes of genes but not others.

### Biogenesis and activity-dependence of Fos ecRNA

**Figure 4:**
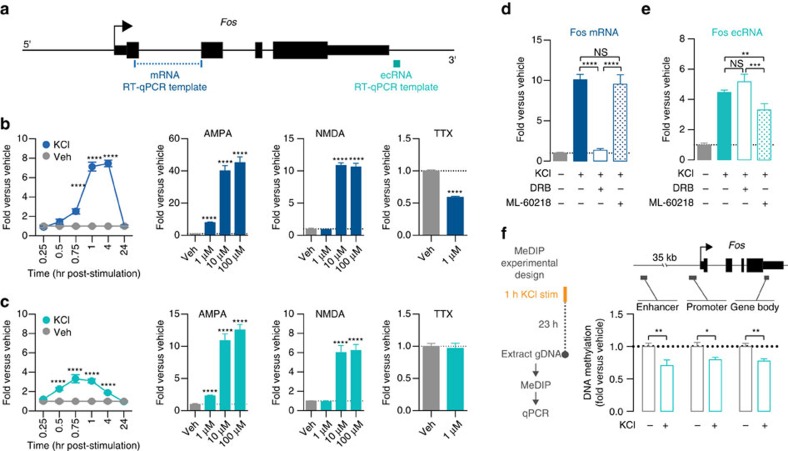
*Fos* ecRNA is differentially responsive to neuronal activation and undergoes unique biogenesis. (**a**) RT-qPCR template locations used to distinguish between *Fos* mRNA and ecRNA. (**b**) Modulation of *Fos* mRNA by KCl, AMPA, NMDA and TTX reveals overall timecourse and activity-dependence of *Fos* gene transcription. *Fos* mRNA is upregulated as soon as 45 min after KCl treatment, and peaks at 4 h following stimulation. AMPA and NMDA treatment (1 h) produced dose-dependent increases in *Fos* mRNA, whereas neuronal silencing with TTX decreased *Fos* mRNA. (**c**) In contrast, *Fos* ecRNA is induced within 30 min of neuronal depolarization with KCl, and peaks at 45 min following KCl treatment. Neuronal stimulation with KCl, AMPA and NMDA induced much lower levels of ecRNA transcription (as compared with mRNA), and neuronal inactivation with TTX did not alter *Fos* ecRNA transcription. (*n*=3–6 per group for KCl experiments, 3 per group for AMPA and NMDA experiments, and 6 per group for TTX experiments). (**d**,**e**) 4-h pretreatment with the RNAPII-dependent transcription inhibitor 5, 6-dichloro-1-beta-D-ribofuranosylbenzimidazole (DRB) had no effect on *Fos* ecRNA but blocked mRNA induction after 1-hour KCl treatment, whereas pretreatment with the RNAPIII inhibitor ML-60218 had no effect on *Fos* mRNA but decreased ecRNA induction after KCl treatment (*n*=6–12 per group; mRNA one-way ANOVA, F_(3,32)_=78.86, *P*<0.0001; ecRNA one-way ANOVA, F_(3,32)_=66.55, *P*<0.0001; Tukey's *post hoc* test for individual comparisons). (**f**) Left, MeDIP experimental design. Right, DNA methylation decreases 24 h after KCl treatment in the enhancer, promoter and gene body of the *Fos* locus (*n*=8, unpaired Student's *t*-test; *t*_16_>2.733 and *P*<0.015 for each comparison). All data are expressed as mean±s.e.m. Individual comparisons, *P<0.05, **P<0.01, ***P<0.001 and ****P<0.0001.

To further dissect the biogenesis, structure and function of ecRNA in neuronal systems, we focused on the ecRNA arising from the activity-regulated *Fos* gene locus (Fig. 4a). Our RNA-seq and RT-qPCR results indicated that a non-polyadenylated transcript arising from the 3′ (post-transcription end site (TES)) *Fos* gene locus was induced by neuronal depolarization with KCl. Therefore, we next examined the time course of *Fos* ecRNA and mRNA induction following neuronal stimulation using RT-qPCR with ecRNA-specific primers (Fig. 4a; Supplementary Data 3). Although KCl increased both *Fos* mRNA and ecRNA, these transcripts had unique induction profiles (Fig. 4b,c). Critically, *Fos* ecRNA (but not mRNA) was significantly elevated as soon as 30 min following KCl stimulation, with maximal ∼3-fold induction at 45 min after stimulation (Fig. 4c). By contrast, *Fos* mRNA levels were not significantly elevated until 45 min following depolarization, and attained maximal levels until 1–4 h after stimulation (Fig. 4b). To examine whether other mechanistically distinct forms of stimulation could induce ecRNAs, neuronal cultures were treated for 1 h with the specific glutamate receptor agonists α-Amino-3-hydroxy-5-methyl-4-isoxazolepropionic acid (AMPA) or N-methyl-D-aspartate (NMDA; Fig. 4b,c). Similar to depolarization with KCl, acute AMPA or NMDA treatment significantly elevated both *Fos* mRNA and 3′ ecRNA, although mRNA induction was consistently higher than ecRNA induction. Finally, to determine whether *Fos* ecRNA levels were also sensitive to neuronal inactivation, neuronal cultures were incubated for 1 h with TTX. Although *Fos* mRNA was substantially reduced by acute TTX treatment, *Fos* ecRNA levels were not altered by neuronal inactivation. Together, these results suggest ecRNA levels in neurons can be activity-dependent and are potentially separate from mRNAs arising from the same gene.

The distinct ecRNA and mRNA synthesis patterns following neuronal activity indicates that ecRNAs are unlikely to be a simple byproduct of conventional mRNA production by RNA polymerases, and also suggests that these transcripts may be controlled by different mechanisms. To examine this possibility, we treated neuronal cultures with selective inhibitors of RNA polymerase II (RNAPII) and RNA polymerase III (RNAPIII) to characterize ecRNA and mRNA biogenesis (Supplementary Fig. 4). Previous studies have demonstrated that *Fos* mRNA, along with many other immediate early genes, is transcribed by RNAPII, which can remain poised at transcription start sites^21^,^22^,^23^. In contrast, RNAPIII transcribes of non-coding elements like transfer and ribosomal RNAs. As expected, we found that basal levels of *Fos* mRNA are sensitive to inhibition of transcription by RNAPII. 4 h treatment with 5, 6-dichloro-1-β-D-ribofuranosyl-1H-benzimidazole (DRB, a pTEFb inhibitor which blocks RNAPII-dependent transcription), dose-dependently decreased *Fos* mRNA (Supplementary Fig. 4a), whereas treatment with an RNAPIII inhibitor (ML-60218) did not alter *Fos* mRNA expression (Supplementary Fig. 4b). In contrast, we found that basal expression of *Fos* ecRNA was sensitive to treatment with either inhibitor, indicating that basal ecRNA transcription may be induced by either RNAPII or RNAPIII. To probe this relationship at another mRNA/ecRNA pair, we repeated this experiment at a second activity-responsive gene (*Atf4*). At this locus, we found a complete dissociation between the effects of RNAPII and RNAPIII inhibition. DRB treatment dose-dependently reduced *Atf4* mRNA, but had no effect on *Atf4* ecRNA. Conversely, RNAPIII inhibition decreased *Atf4* ecRNA but did not decrease *Atf4* mRNA (Supplementary Fig. 4).

Together, these results further suggest that ecRNA is not simply a non-polyadenylated byproduct of normal gene transcription. However, steady-state transcript levels are the result of complex dynamics between transcript synthesis and degradation. To further examine activity-dependent synthesis of *Fos* mRNA and ecRNA, we pre-treated neuronal cultures with RNAPII or RNAPIII-dependent transcriptional inhibitors before KCl-induced depolarization (Fig. 4d,e). Here, we found that while KCl-induced increases in *Fos* mRNA were prevented by pretreatment with DRB, RNAPII-dependent transcription inhibition was unable to block increases in *Fos* ecRNA. In contrast, ML-60218 significantly attenuated *Fos* ecRNA increases following KCl (but did not alter KCl-induced *Fos* mRNA), suggesting that at least part of the *Fos* ecRNA induction in response to neuronal depolarization is driven by RNAPIII transcription. In agreement with this, RNAPIII binding at the *Fos* promoter was significantly increased following KCl stimulation, as detected using chromatin immunoprecipitation (ChIP) for the RNAPIII subunit RPC32 (Supplementary Fig. 4c). Thus, these findings reveal a critical dissociation between RNAPII and RNAPIII driven transcription at the *Fos* locus, and further confirm the independence of activity-dependent *Fos* ecRNA and *Fos* mRNA synthesis.

Given the genome-wide relationship between DNA methylation and ecRNA expression, we hypothesized that *Fos* ecRNA increases following KCl stimulation might be associated with subsequent *Fos* hypomethylation. To test this, we examined DNA methylation levels at three sites relative to the *Fos* gene (distal upstream enhancer, proximal promoter and gene body) using methylated DNA immunoprecipitation (MeDIP) 24 h after KCl-induced neuronal depolarization. As shown in Fig. 4f, we observed decreases in DNA methylation at each of these locations following KCl treatment, which is consistent with the genome-wide relationship between ecRNA and DNA methylation.

### ecRNAs directly bind to DNMT3a and prevent methylation

**Figure 5:**
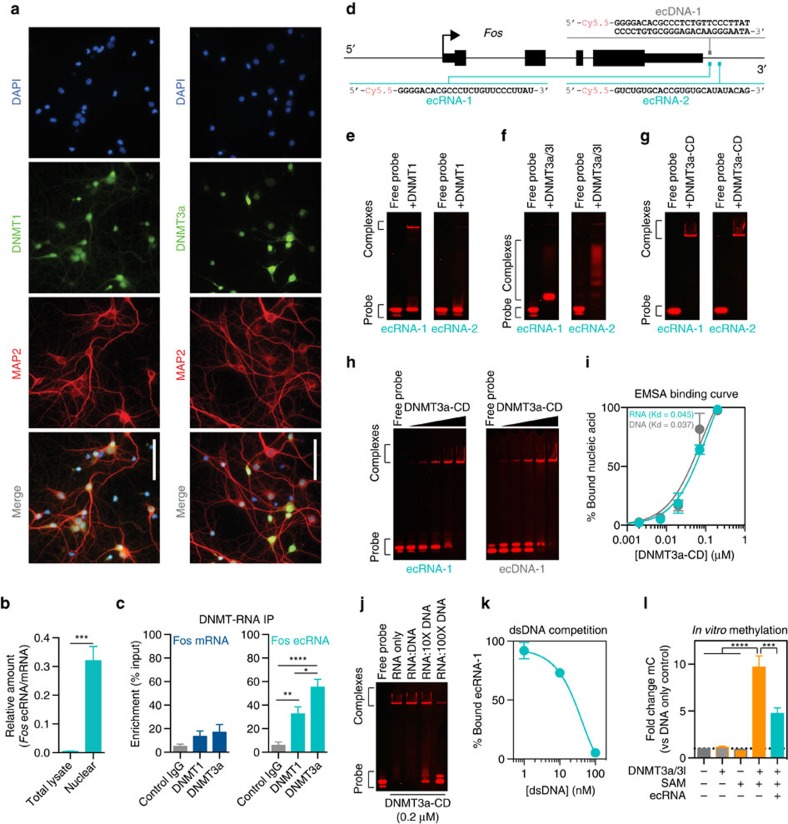
*Fos* ecRNA interacts with DNA methyltransferases and blocks DNA methylation. (**a**) Immunostaining reveals nuclear localization of DNMT1 and DNMT3a in neuronal cultures. Cell nuclei are stained with 4,6-diamidino-2-phenylindole (DAPI), and neurons are marked by MAP2 (microtubule-associated protein 2). Scale bar, 50 μm. (**b**) *Fos* ecRNA/mRNA comparison in total neuronal lysate and nuclear fraction (separated during RNA-IP; *n*=4 per group; unpaired Student's *t*-test, *t*_6_=6.301, *P*=0.007). (**c**) *Fos* ecRNA, but not mRNA, immunoprecipitates with anti-DNMT1 or DNMT3a antibodies but not control IgG (*n*=4–6 per group; ecRNA one-way ANOVA, F_(2,15)_=20.53, *P*<0.0001; Tukey's *post hoc* test for individual comparisons). (**d**) Locations of synthetic RNA and DNA oligonucleotides used in mobility shift assays. (**e**) Electrophoretic mobility shift assay reveals only slight binding of ecRNA probes (1 nM) to recombinant DNMT1 (0.2 μM). RNA/DNMT complexes are evident as low-mobility band on native PAGE gel following incubation with DNMT protein. (**f**) Synthetic ecRNA probes (1 nM) bind recombinant DNMT3a/DNMT3l protein (0.2 μM). (**g**) Complete binding of synthetic ecRNA probes (1 nM) to truncated recombinant DNMT3a protein (0.2 μM) containing only catalytic domain (DNMT3a-CD). (**h**) Incubation of ecRNA-1 or ecDNA-1 probes (1 nM) with escalating concentrations of DNMT3a-CD (0.002–0.2 μM). (**i**) DNMT3a-CD binds equally to RNA and double-stranded DNA with the same primary sequence. Binding affinity (Kd values; derived from non-linear, one-site regression analysis of complete concentration curve) for RNA and dsDNA was not significantly different (*n*=2 replicates; comparison of Kd, F_(1,22)_=0.47, *P*=0.49). (**j**) Competition assay between ecRNA-1 (1 nM) and unlabelled ecDNA-1 probes (1–100 nM) shows intact RNA/DNMT3a-CD complexes even with 10-fold higher concentrations of DNA. (**k**) Quantification of dsDNA competition assay. (**l**) Co-incubation of DNMT3a/3l protein and the methyl donor SAM results in cytosine methylation at dsDNA (3.42 nM) from the *Fos* promoter. Methylation was significantly inhibited by addition of ecRNA (5.16 μM). For (**l**) *n*=4 per group, one-way ANOVA, F_(4,15)_=40.25, *P*<0.0001; Tukey's *post hoc* test for individual comparisons. All data are expressed as mean±s.e.m. Individual comparisons, *P<0.05, **P<0.01, ***P<0.001 and ****P<0.0001.

Next, we sought to examine how ecRNAs might regulate DNA methylation status. As previous studies suggest that DNA methyltransferases bind to non-coding RNA species^15^,^16^,^17^, we hypothesized that *Fos* ecRNA controls DNA methylation at this gene by interacting with DNA methyltransferases. To examine this possibility, we first verified nuclear expression of the major DNA methyltransferases (DNMT1 and DNMT3a) in our neuronal cultures using IP-grade DNMT antibodies and MAP2 co-staining to identify neurons (Fig. 5a). This staining revealed mostly nuclear localization of DNMTs. Likewise, PolyA+ RNA-seq revealed that *Dnmt1* and *Dnmt3a* are the most abundant DNMT transcripts in cortical neuronal cultures (*Dnmt1* FPKM mean=17.18; *Dnmt3a* FPKM mean=14.57; *Dnmt3b* FPKM mean=0.79). To determine whether *Fos* ecRNA might interact with DNMTs, we performed an RNA immunoprecipitation (RIP) with antibodies for DNMT1 and DNMT3a. As part of the RNA-IP protocol, we gently permeabilized the cellular membrane, which allowed for nuclear isolation and collection. In non-IP (input control) samples, we found that ecRNAs were significantly enriched as compared with total lysate fractions, indicative of nuclear localization (Fig. 5b). In IP samples, RT-qPCR was used to detect the presence of ecRNAs bound to DNMTs, and revealed significant enrichment of *Fos* ecRNA on DNMT1 and DNMT3a, as compared with a normal immunoglobulin-G (IgG) control (Fig. 5c). In contrast, *Fos* mRNA was not significantly enriched in DNMT-RIP samples, demonstrating selectivity of DNMT binding to extra-coding transcripts arising from the *Fos* locus.

These results are the first indication that ecRNAs bind to the *de novo* DNA methyltransferase DNMT3a. To examine this interaction in more detail, we performed a series of *in vitro* DNMT binding assays with synthetic, fluorescently labelled 25-nucleotide RNA and double-stranded DNA probes based on sequences from the *Fos* ecRNA locus (Fig. 5d). Interaction between synthetic RNAs and DNMTs was detected using an electrophoretic separation mobility shift assay (EMSA; Fig. 5e–j). For both RNAs examined (ecRNA-1 and ecRNA-2), we observed slight binding to DNMT1 (Fig. 5e) and robust binding to recombinant DNMT3a either in complex with DNMT3l (which prevents DNMT3a aggregation and allows greater electrophoretic mobility; Fig. 5f), or the isolated catalytic domain of DNMT3a (Fig. 5g). RNA did not bind other proteins with similar size or charge, and binding was maintained with the addition of the non-specific competitor poly-dI-dC (see Methods section and Supplementary Fig. 5c). To directly compare DNMT3a binding to RNA and DNA with the same primary sequence, we conducted complete binding curves to derive the affinity of DNMT-nucleic acid binding (Fig. 5h). Strikingly, ecRNA sequences bound the catalytic domain of DNMT3a with similar affinity as double-stranded DNA, with Kd values in the low nanomolar range (Fig. 5i). Moreover, a competition assay (Fig. 5j,k) revealed that over 20-fold excess of dsDNA was required to reduce ecRNA-DNMT binding by half, suggesting robust ecRNA-DNMT3a interactions even in the presence of double-stranded DNA.

Next, we used an *in vitro* DNA methylation assay to test whether the presence of ecRNA could block the ability of DNMTs to methylate double-stranded DNA. Although either DNMT3a/3l protein or the methyl donor S-adenosyl methionine (SAM) alone was insufficient to methylate an isolated dsDNA fragment from the *Fos* promoter region, co-incubation of DNMT and SAM induced robust methylation of the target DNA (Fig. 5l). Consistent with the ability of ecRNA to bind DNMT3a directly at the catalytic domain, and with the genome-wide relationship between ecRNA and DNA methylation, we found that addition of ecRNA to the *in vitro* methylation reaction was sufficient to impair methylation of the *Fos* promoter target DNA.

The ability of ecRNAs to form step-loop structures has been shown to mediate interactions between ecRNAs and DNMT1 (ref. 15). To determine whether ecRNA-DNMT3a interactions were also dependent on stem-loop structure, we designed ‘mutant' synthetic RNAs that possessed identical base composition as synthetic ecRNA-1 and ecRNA-2, but lacked a predicted secondary structure (Supplementary Fig. 5). DNMT3a-CD interactions were not impaired in mutant RNAs lacking predicted stem-loop secondary structure (Supplementary Fig. 5a,b). These results suggest that stem-loop structure is not required for ecRNA-DNMT3a interactions.

### Fos ecRNA reduction alters methylation and gene expression

**Figure 6:**
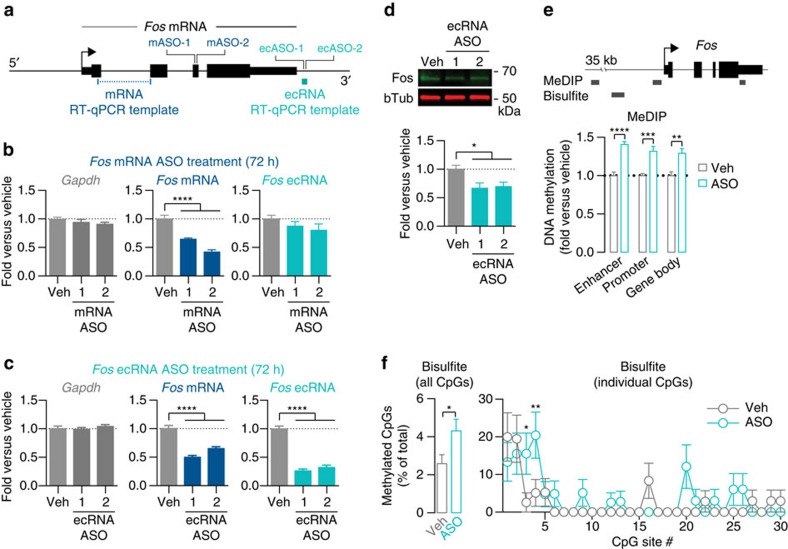
*Fos* ecRNA controls *Fos* gene methylation. (**a**) Anti-sense oligonucleotide (ASO) target locations for *Fos* mRNA and ecRNA. (**b**) *Fos* mRNA ASOs decreased mRNA expression with no significant effect on ecRNA (*n*=8 per group; one-way ANOVA for *Fos* mRNA, F_(2,21)_=42.96, *P*<0.0001; Tukey's *post hoc* test for individual comparisons.). (**c**) *Fos* ecRNA ASOs decreased both mRNA and ecRNA (*n*=8 per group; one-way ANOVAs for *Fos* mRNA and ecRNA, F_(2,21)_=36.43 and 95.48, respectively, *P*<0.0001; Tukey's *post hoc* test for individual comparisons). (**d**) *Fos* ecRNA knockdown reduces *Fos* protein quantified by immunoblotting (*n*=6 per group; one-way ANOVA, F_(2,15)_=5.438, *P*=0.0168). (**e**) *Fos* ecRNA knockdown (72 h ASO treatment) resulted in increased enhancer, promoter and gene body methylation as measured by MeDIP (*n*=4 per group; two-way ANOVA main effect of ASO, F_(1,18)_=71.60, *P*<0.0001, Sidak's *post hoc* test for multiple comparisons). (**f**) Bisulfite sequencing following *Fos* ecRNA knockdown confirmed significant promoter hypermethyation (left, average of all CpG sites; 41–45 individual clones/group, *χ*^2^ test, *z*=2.213, *P*=0.0269), driven largely by specific hypermethylation at two distinct CpG sites (right; methylation status between treatment groups compared with a two-way ANOVA with Sidak's *post hoc* tests adjusted for multiple comparisons at individual CpG sites). Data are expressed as mean±s.e.m. Individual comparisons, *P<0.05, **P<0.01, ***P<0.001 and ****P<0.0001.

Overall, our results suggest a model in which ecRNA species generated from a protein-coding gene can bind to DNA methyltransferases to inhibit DNA methylation at the promoter of that gene. This model is intriguing in that it indicates that protein-coding transcription could be dynamically tuned by manipulating ecRNA status at a given gene. To test this prediction, we designed chemically modified anti-sense oligonucleotides (ASOs) that selectively target *Fos* 3′ ecRNA (at locations at least 100 bp from the mRNA TES) or *Fos* mRNA (in exon 3) (Fig. 6a; Supplementary Data 3). As our model predicted, ASOs that targeted the *Fos* 3′ ecRNA significantly reduced both the ecRNA and the mRNA, while also significantly decreasing Fos protein levels (Fig. 6c,d). In contrast, ASOs targeting *Fos* mRNA specifically decreased mRNA levels, but did not alter *Fos* ecRNA (Fig. 6b). To examine how ASO-mediated ecRNA degradation affected DNA methylation status at the *Fos* gene, we assayed DNA methylation levels using MeDIP (at the *Fos* enhancer, promoter and gene body; Fig. 6e) and bisulfite sequencing (at the *Fos* promoter; Fig. 6f). ASO-mediated *Fos* ecRNA degradation increased DNA methylation levels as assayed both by MeDIP and bisulfite sequencing. Overall, bisulfite analysis revealed that individual CpGs in the *Fos* promoter were 1.7 times more likely to be methylated following ASO treatment than in vehicle-treated controls, and this effect was largely driven by significant ASO-induced hypermethylation at two distinct CpG sites (Fig. 6f). In contrast, treatment with a *Fos* mRNA ASO that resulted in comparable degradation of *Fos* mRNA levels without altering ecRNA expression did not significantly affect *Fos* promoter methylation status as measured by MeDIP (Supplementary Fig. 6). Finally, to examine the potential stability of ASO-mediated ecRNA knockdown, we treated neuronal cultures with *Fos* ecRNA ASO for up to 7 days (Supplementary Fig. 6). This experiment demonstrated enduring ecRNA knockdown for up to 1 week after ASO treatment. Furthermore, consistent with a mechanism by which DNA methylation accumulates at the *Fos* locus with continued ecRNA knockdown, we observed a progressive decline in *Fos* mRNA expression with prolonged treatment. Together, these results suggest that loss of ecRNA at a gene locus induces promoter hypermethylation and transcriptional silencing of the associated gene.

### Fos ecRNA is required for long-term memory formation

Early transcriptional induction from the *Fos* gene locus after neuronal activity is a common feature across neuronal subtypes and functionally defined regions of the central nervous system (CNS). Fos protein forms a heterodimer with Jun family members to generate an AP-1 transcription factor, which regulates gene expression networks in response to stimulation^24^,^25^,^26^. Although baseline levels of *Fos* mRNA are relatively low in adult animals^27^,^28^, *Fos* mRNA transcription is upregulated following multiple types of memory formation^4^,^9^,^29^. Further, mice with conditional deletion of the *Fos* gene in the adult CNS exhibit impaired hippocampal synaptic long-term potentiation and impaired performance on hippocampus-dependent long-term memory tasks, including contextual fear conditioning^30^.

**Figure 7:**
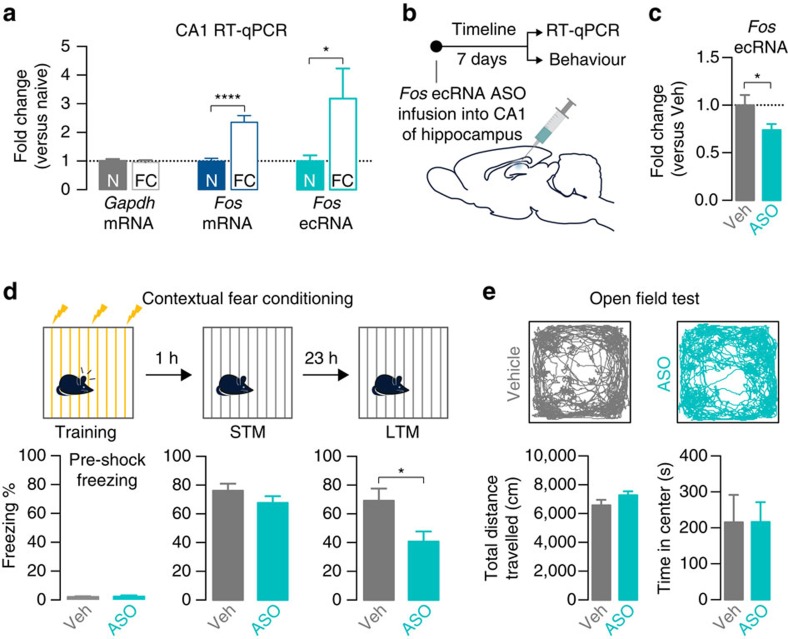
*Fos* ecRNA is induced by behavioural experience and modulates memory formation. (**a**) Contextual fear conditioning is associated with increased *Fos* mRNA and *Fos* ecRNA in the CA1 subregion of the hippocampus, as compared with experimentally naïve controls (N=naïve, FC=fear conditioned; *n*=16 per group; Mann—Whitney *U* test, *U*=15 and *P*<0.0001 for *Fos* mRNA, *U*=73 and *P*=0.038 for *Fos* ecRNA). Area CA1 was subdissected from total hippocampus 1 h following contextual fear conditioning. (**b**) Experimental timeline for *Fos* ecRNA ASO experiments *in vivo*. (**c**) *Fos* ecRNA ASO treatment decreased *Fos* ecRNA expression in the CA1 of the hippocampus (*n*=8–9 per group, unpaired Student's *t*-test, *t*_15_=2.173, *P*=0.0462). (**d**) Top, contextual fear conditioning design. Bottom, *Fos* ecRNA ASO treatment impaired long-term memory but did not alter baseline freezing or short-term memory (*n*=8–9 per group; STM unpaired Student's *t*-test, *t*_15_=1.245, *P*=0.23; LTM unpaired Student's *t*-test, *t*_15_=2.640, *P*=0.0186). (**e**) Open field test. Top, representative traces showing animal location during 30 min test session. *Fos* ecRNA knockdown did not affect total distance travelled (bottom left; unpaired Student's *t*-test, *t*_14_=1.517, *P*=0.1514) or time spent in the center of an open field test (bottom right; unpaired Student's *t*-test, *t*_14_=0.005, *P*=0.99; *n*=8 per group). All data are expressed as mean±s.e.m. Individual comparisons, *P<0.05 and ****P<0.0001.

To examine the potential role for *Fos* ecRNA in long-term memory formation, we trained rats in a standard contextual fear conditioning task in which three 1 mA footshocks were paired with a novel context. One hour after training, the CA1 of the hippocampus was removed. RT-qPCR using RNA extracted from the CA1 revealed a significant increase in both *Fos* mRNA and *Fos* ecRNA as compared with an experimentally naïve group that did not undergo fear conditioning (Fig. 7a). This result suggests that ecRNA species can be induced in the adult brain in an experience-dependent manner. To examine the function of *Fos* ecRNA in memory formation, we stereotaxically infused either vehicle or *Fos* ecRNA ASOs into the dorsal CA1 subregion of the hippocampus (Fig. 7b), followed by either RT-qPCR to verify *Fos* ecRNA knockdown (Fig. 7c), or by behavioural testing (Fig. 7d,e). Consistent with *in vitro* results, *Fos* ecRNA ASO treatment produced a significant decrease in *Fos* ecRNA levels in the CA1 of the hippocampus up to 7 days after infusion (Fig. 7c). Seven days after ASO treatment, rats underwent contextual fear conditioning followed by short-term (1 h post-training) and long-term (24 h post-training) tests for memory formation. *Fos* ecRNA ASOs did not alter baseline (pre-shock) freezing during initial the initial training session or short-term contextual fear memory. In contrast, *Fos* ecRNA knockdown resulted in a significant deficit in freezing responses observed on re-exposure to the conditioning chamber after 24 h (Fig. 7d), indicative of a selective impairment in long-term contextual fear memory formation. Importantly, ASO treatment did not alter performance in an open field test (Fig. 7e), suggesting that deficits in contextual fear memory were not due to altered locomotor activity or differences in anxiety-like behaviours.

## Discussion

In this study, we provide novel genome-wide evidence for an association between ecRNA species and DNA methylation states in neuronal systems. We find that in the absence of ecRNA production, genes tend to possess methylated promoters and are silent. In contrast, genes that generate ecRNAs possess demethylated promoters and are actively transcribed. Using unbiased mathematical clustering, we observed that this relationship is preserved at actively transcribed neuronal genes, including genes modulated by neuronal activity and genes implicated in developmental and neurodegenerative disorders. This genome-wide characterization provides a key initial step in understanding how ecRNAs might regulate epigenetic states within neurons, and also provides insight into the biological context of this phenomenon across gene classes.

These results are consistent with previous observations that the epigenome is highly dynamic in the adult brain, and reveal one of the first potential mechanisms for activity- and experience-dependent alterations in cytosine methylation status at specific gene loci. In addition to providing a genome-wide characterization of activity-regulated transcriptional dynamics of both polyadenylated and non-polyadenylated RNA species in neurons, our results provide a detailed analysis of ecRNAs arising from the *Fos* gene locus. We show that *Fos* ecRNAs interact directly with DNMTs, that DNA methylation states at *Fos* are altered following ecRNA increases, that *Fos* ecRNAs undergo distinct synthesis and regulation from *Fos* mRNA, and that knockdown of *Fos* ecRNA increases DNA methylation and represses *Fos* mRNA. Furthermore, we reveal that *Fos* ecRNA is induced by the memory-forming experiences, and is necessary for expression of contextual fear memory. These findings highlight a potential role for ecRNAs in cognitive function, and further illustrate the dynamic link between active non-coding RNA transcription and epigenetic regulation in neurons.

One challenge in genome-scale identification and quantification of ecRNA species is that non-polyadenylated RNA fractions could also contain nascent unspliced pre-mRNA that has not yet been polyadenylated. Although previous reports have revealed widespread co-transcriptional splicing in neurons^31^, it is likely that nascent mRNA transcripts contributed in some way to the intronic ecRNA quantification strategy employed here. Though this issue is difficult to resolve using standard whole-genome sequencing approaches, more detailed single-molecule sequencing could be employed to capture longer transcripts and thus provide more complete disambiguation of ecRNA and pre-mRNA transcripts. Critically, this analysis could also facilitate identification of ecRNA transcript start and end sites, as well as enriched sequence motifs that might confer ecRNAs with targeted DNA or protein recognition properties. Nevertheless, our characterization of ecRNA from the 3′ end of the *Fos* gene locus is in agreement with previous observations that ecRNAs are regulated distinctly from overlapping mRNA and contribute to gene-specific DNA methylation patterns^15^. Based on our genome-wide gene expression and DNA methylation data, we hypothesize that ecRNA transcripts arising from other genes also possess differential biosynthesis and regulation. However, although our results suggest that activity-dependent induction of the *Fos* ecRNA is controlled by RNAPIII, it remains unclear if this is the case at other ecRNA transcription sites. Future studies will be required to explore differential polymerase contributions to transcription of functionally defined non-coding RNA subclasses.

Overall, our results add to an emerging appreciation for non-coding RNA species in epigenetic and transcriptional modulation^32^, including the ability to act as decoy molecules or scaffolds for transcriptional regulators, target specific modifiers to a unique DNA locus, and directly bind to DNA itself. We propose that ecRNAs directly interact with the DNMT catalytic domain to block the ability of DNMTs to target and silence overlapping genes. One prediction of this model is that ecRNAs would need to remain co-localized with the parent gene following synthesis to provide locus-specific DNMT regulation. Although how this occurs is presently unknown, there are at least two distinct possibilities. One is that ecRNAs could possess extremely short half-lives (as has been demonstrated for enhancer RNAs (ref. 22)), in effect restricting function to a local chromatin environment surrounding the overlapping gene. However, a second and potentially more intriguing possibility is that ecRNAs could form a triplex structure with DNA (ref. 33) that serves the dual purpose of stabilizing the ecRNA and providing locus-specific anchor for interference of DNMT activity^15^. Regardless of the specific mechanism, characterizing the nature of this process will be important for understanding how individual cytosine nucleotides can be targeted for developmental or activity-dependent epigenetic reprogramming^1^,^8^.

Our results also suggest the possibility that differential regulation of ecRNA species may serve as a unique means for endogenous control of the neuronal epigenome. Given that many neuropsychiatric and neurological diseases are associated with chronic alterations in specific gene products^34^,^35^,^36^, we speculate that ecRNA targeting may provide an attractive therapeutic approach that could deliver gene-specific epigenetic reorganization to alleviate longstanding epigenetic pathologies. Indeed, co-opting endogenous mechanisms for epigenetic control represents an entirely new layer of therapeutic possibility, and could include the de-repression of methylated genes or the silencing of actively transcribed genes^37^. Moreover, because cytosine methylation can be self-perpetuating^38^,^39^, ecRNA-targeted alterations in gene methylation could conceivably persist beyond the initial triggering stimulus to generate enduring transcriptional regulation.

## Methods

### Cultured neuron experiments

Primary rat cortical neuronal cultures were generated from embryonic day 18 rat cortical tissue as described previously^4^. Cell culture wells were coated overnight at 37 °C with poly-L-lysine (50 μg ml^−1^) and rinsed three times with diH_2_O. Dissected cortices were incubated with papain for 20 min at 37 °C. After rinsing in Hank's Balanced Salt Solution, a single-cell suspension of the tissue was re-suspended in Neurobasal media (Invitrogen) by trituration through a series of large to small fire-polished Pasteur pipets. Primary neuronal cells passed through a 70-μM cell strainer were plated on poly-lysine coated culture wells. Cells were grown in Neurobasal media plus B-27 and L-glutamine supplement (complete Neurobasal media) for 8–11 days *in vitro* in a humidified CO_2_ (5%) incubator at 37 °C.

At 8–11 days *in vitro*, neuronal cultures were treated as described. For KCl stimulation experiments, 25 μl of 1 M KCl (Sigma) or vehicle (neurobasal media alone) was added to cell culture wells to achieve a final concentration of 25 mM KCl. Cells were incubated with KCl for described time points before RNA extraction. For TTX inactivation experiments, cells were treated with 1 μM TTX (Tocris Bioscience) in neurobasal media for the described time points before RNA extraction. For AMPA and NMDA experiments, neuronal cultures were treated with the described concentrations of S-AMPA or NMDA (Sigma) for 1 h. S-AMPA and NMDA were diluted in sterile water and added to cultures at a volume of 10 μl. 10 μl sterile water was added as a vehicle control. For experiments involving RNAP inhibitors, cultures were treated for 4 h (Fig. S4) or 4 h followed by a 1 h, 25 mM KCl stimulation (Fig. 4d,e). The RNAPII-dependent transcriptional inhibitor DRB and the RNAPIII inhibitor ML-60218 (Sigma) were dissolved to a 20-mM stock solution in 100% cell culture grade DMSO (Sigma) and diluted in neurobasal media to described experimental concentrations. Vehicle-treated cells received equal concentrations of DMSO in neurobasal media. At a minimum, all cell culture experiments were performed in triplicate.

### RNA-seq

RNA-seq experiments using two biological replicates per condition were carried out at the Hudson Alpha Genome Services Laboratory. RNA was extracted, DNase-treated and purified (RNeasy, Qiagen). Two distinct RNA libraries were generated. Polyadenylated (PolyA+) RNA was captured with the NEBNext Poly(A) mRNA Magnetic Isolation Module. The remaining non-polyadenylated (PolyA−) underwent ribosomal RNA depletion (NEBNext rRNA depletion kit). 2 μg of total RNA underwent quality control (Bioanalyzer; all RIN values >9.5), and was prepared for directional RNA sequencing at Hudson Alpha using NEBNext reagents (New England Biolabs) according to manufacturer's recommendations with minor modifications (including the use of custom library adaptors and indexes). PolyA+ and PolyA− RNA libraries underwent sequencing (50 bp paired-end directional reads; ∼25 M reads/sample) on an Illumina sequencing platform (HiSeq2000).

### RNA-seq data analysis

Raw paired-end sequenced reads were quality controlled, filtered for read quality (FASTX toolkit, Galaxy) and aligned to the rat Rn5 genome sequence in Galaxy using Tophat v1.4.0 (with custom settings –p 8 –r 175). Overall, we obtained ∼130 million total paired-end mapped PolyA+ reads and ∼118 million mapped PolyA− reads from six independent biological replicates. Genome-aligned sequenced reads were examined in Seqmonk software release v0.28.0 (Babraham Institute), using Ensembl release v70 gene and feature annotations with modifications to update 3′UTR annotation (as described below). For all sequencing analysis, we filtered gene annotations to exclude Ensembl predicted/model genes and short genes (<1 kb), leaving 17,719 genes for characterization. For independent PolyA+ samples, transcript expression levels were determined by computing the FPKM. Gene expression differences between groups (Veh versus KCl) were calculated in Seqmonk using the Intensity Difference function, correcting for multiple testing. Statistical significance was assessed using Student's *t*-tests and a false-discovery rate of 0.05. Comparisons and analysis in Figs 1 and 3, and S1 was performed with data sets from unstimulated cells (vehicle group).

To characterize the genome-wide expression of ecRNA-like transcripts, we computed FPKM values at every gene in three distinct locations: (1) 5′, pre-transcription start site (−1,000 bp upstream to TSS), (2) introns and (3) 3′ post-TES (TES to 2,000 bp past TES). For this analysis, we included only PolyA- reads from the ‘sense' strand of a given gene, to exclude anti-sense transcripts. For genes containing multiple introns, composite (mean) FPKM values were computed to quantify intronic transcription across the gene. Intronless genes did not receive a value for the ‘intron' quantification. Pre-TSS, intronic and post-TES FPKM values were averaged to obtain an ‘ecRNA index', which provided an overall quantification for ecRNA-like transcription from every gene.

### *De Novo* 3′UTR annotation.

Recent reports have revealed robust 3′UTR extension and lengthening of neuronal genes^40^,^41^,^42^, which are frequently not annotated in available rat genome assemblies. Given that 3′UTR extensions could impair accurate quantification of 3′ ecRNAs across the genome, we performed a *de novo* annotation of 3′UTR end sites throughout the genome. Our 3′UTR identification pipeline (based on^40^) utilized a merged library consisting of ∼130 million total paired-end mapped PolyA+ reads from vehicle, KCl and TTX treated cells. We first identified contiguously transcribed regions of the genome using Seqmonk software, with a fivefold (21 × read depth) coverage cutoff. To identify only meaningful 3′UTR extensions, we limited our annotation to regions at least 200 bp in length, and merged contiguous regions separated by <150 bp. Given that our libraries were directional, we separated forward and reverse strands for this analysis and considered transcripts arising from each strand separately. Genome-wide, this analysis identified 52,651 regions of contiguous transcription. To identify 3′UTR extensions, we filtered this list for regions that overlapped existing transcription end annotations for our entire gene set, which identified 5,801 potential candidate 3′UTR extensions. Following additional filtering to remove regions that overlapped the start site of adjacent genes, regions from internally transcribed genes and potential anti-sense transcripts, our list of 3′UTR extensions was reduced to 4,926 genes. Visual inspection and overall global alignments indicated precise 3′UTR annotation following this pipeline, and correctly identified many previously observed 3′UTR extensions, including the 18.7 kbp 3′UTR extension at *Grin2b*, the 16.3 kbp extension at *Ntrk3*, as well as extensions at *Nedd4l*, *Dnajc15* and *Hmbox1* (ref. 40). Overall, this analysis added ∼8.3 Mbp to the annotated transcriptome, with an average extension of 1,703 bp at the 4,926 genes with new 3′UTR termination annotations. All 3′ecRNA characterizations were performed with these updated annotations.

Even with corrected 3′UTR annotations, it is still possible that ecRNA estimates could be skewed due to another type of assembly fault (for example, mislocated exons or exons expressed only in neurons), the presence of overlapping sense-strand genes, or the inclusion of inactive genes that bias genome-wide correlations. To address each of these concerns, we performed an additional analysis designed to estimate the normalized level of ecRNA, compared with PolyA+ RNA from the same loci. This analysis revealed the same relationship between ecRNA levels and promoter DNA methylation (Supplementary Fig. 7).

### MBD-seq and capture verification

Genome-wide DNA methylation patterns were quantified using MBD protein capture (MBD-capture; MethylMiner Kit, Invitrogen), followed by next-generation sequencing. DNA from unstimulated neuronal cultures was extracted, RNase treated and purified (DNeasy, Qiagen). 2 μg of genomic DNA was sonicated to 200–400 bp (Bioruptor Pico, Diagenode). Methylated DNA was collected with recombinant MBD2 protein/biotin complex, which was purified using streptavidin-coated magnetic beads (Invitrogen). DNA sequencing was performed at Hudson Alpha using NEBNext reagents (New England Biolabs) according to manufacturer's recommendations with minor modifications (including the use of custom library adaptors and indexes). DNA libraries were quantified with the Kapa Library Quant Kit (Kapa Biosystems), and underwent sequencing (25 M total 50 bp single-end reads) on an Illumina sequencing platform (HiSeq2000). We sequenced two biological replicates as well as an input (non-IP) control for normalization. To ensure that MBD-capture resulted in the specific enrichment of methylated DNA, we performed control reactions in which gDNA was spiked with synthetic methylated and non-methylated DNA fragments (1 pg each, Methyl Miner kit, Invitrogen) before immunoprecipitation with recombinant MBD2. Methylated and non-methylated DNA capture was quantified via RT-qPCR with primers specific for each synthetic sequence, using both the captured (MBD2-bound) and unbound fractions. Our results demonstrated robust enrichment (∼600-fold) of methylated DNA fragments in the captured sample, and equally robust depletion of methylated DNA in the unbound fraction (Supplementary Fig. 2b).

### MBD-seq data analyses

Raw single-end sequenced reads were quality controlled, filtered for read quality (FASTX toolkit, Galaxy) and aligned to the rat genome (Rn5 assembly) in Galaxy using Bowtie. Overall, we obtained ∼44 M mapped single-end reads from MBD-IP samples, and ∼20 M mapped reads from an input control sample. Genome-aligned sequenced reads were examined using SeqMonk (Babraham Institute). For each sample, methylation levels were examined relative to gene elements using the built-in analysis pipelines. Promoter methylation was quantified relative to the TSS by computing the reads per million for a 5-kb window surrounding the TSS (−2.5 to 2.5 kb). To remove potential confounds arising from DNA sequence mappability, all data were corrected by subtracting reads per million values from the input (non-IP) control. This pipeline resulted in highly reproducible methylation quantification at both gene bodies (Supplementary Fig. 2c) and gene promoters (Supplementary Fig. 2d). Additional analysis based on CpG identification (Supplementary Fig. 3) was performed using annotated CpG island boundaries.

### Hierarchical clustering and gene ontology

Hierarchical clustering analysis and dendrogram generation (Fig. 3) was performed in Matlab using the clustergram toolbox. Clustering was performed using percentile scores instead of raw data to equalize data ranges between columns. Each row was sorted based on Euclidean distance metrics and average linkage to generate a hierarchical tree. To integrate this information with existing data sets, gene lists corresponding to autism spectrum risk genes, Alzheimer's disease risk genes, or genes altered in a mouse model of Alzheimer's disease (Fig. 3b) were obtained from published reports^34^,^43^,^44^.

Gene ontology analysis of gene clusters identified in using this approach was performed using the ClueGO plugin in Cytoscape^45^. Enrichment analysis was conducted using a reference set of all 17,719 genes. Significantly enriched biological process terms (hierarchy levels 8–15) containing at least 10% of genes in each category were identified using a Benjamini–Hochberg false-discovery rate and *α*=0.001. For comparison to previously published data sets, human and mouse gene names were mapped onto rat gene orthologues according to gene symbols. Only genes with identical rat, mouse and human symbols are shown here, resulting in the exclusion of some genes that were found in original lists but did not map to rat gene symbols.

### RNA quantification with RT-qPCR

Total RNA was extracted using the RNeasy Mini kit (Qiagen) following the manufacturer's instructions. All samples were treated with DNase1 during column purification to remove contaminating DNA. RNA was reverse transcribed using the iScript RT-PCR kit (Bio-Rad). PCR amplification was performed in triplicate (cell culture experiments) or with six replicates (*in vivo* experiments) using a CFX96 real-time PCR system (Bio-Rad) at 95 °C for 3 min, followed by 40 cycles of 95 °C for 10 s and 58 °C for 30 s, followed by real-time melt analysis to verify product specificity. *Gapdh* (cell culture experiments) or *Actb* (*in vivo* experiments) were used as internal controls for normalization using the ΔΔCt method^46^.

### Chromatin immunoprecipitation

RNAPIII binding at specific genomic loci was assayed using available ChIP protocols with minor modifications^47^,^48^. Briefly, cultured cells were treated as described and immediately fixed in 1% paraformaldehyde, washed in phosphate-buffered saline (PBS), lysed and then sonicated (Bioruptor Pico, Diagenode) to shear DNA to 200–500 bp fragments. Sheared, cross-linked DNA was incubated with 5 μl RNAPIII antibody (RPC32 subunit, #sc-21754, Santa Cruz Biotechnology) and 25 μl protein-A coated magnetic beads (Invitrogen) overnight, washed sequentially in low salt, high salt, LiCl and tris-EDTA (TE) buffers, and then incubated for 2 h at 65 °C in TE buffer containing 1% SDS and proteinase K solution (Qiagen) to reverse crosslinks. Following magnetic removal of protein-A coated beads, extracted DNA was then purified (Qiagen DNA Mini Spin Column), and RNAPIII binding levels at the *Fos* gene promoter were assayed via qPCR as described above. Ct values for IP samples were normalized to unprocessed (input) DNA, which was not incubated with RNAPIII antibody.

### DNMT/MAP2 immunostaining

To verify expression of DNMT isoforms in neuronal cultures, we performed immunolabeling for DNMT1 and DNMT3a. After removal of neuronal culture media, cells were washed with PBS and incubated at room temperature for 20 min in freshly prepared 4% paraformaldehyde in PBS. After fixation, cells were washed three times with PBS and neuronal membranes were permeabilized with PBS containing 0.25% Triton X-100 for 15 min at room temperature. Cells were then washed three times in PBS, blocked for 1 h (10% Thermo Blocker bovine serum albumin (BSA) #37525, 0.05% Tween-20, and 300 mM glycine in PBS) and co-incubated with DNMT (1:1,000 in PBS with 10% Thermo Blocker BSA #37525, Abcam anti-DNMT1 (ab87656) or anti-DNMT3a (ab2850)) and MAP2 antibodies (1:250, Anti-MAP2 Alexa Fluor 555 Conjugate, Invitrogen) at 4 °C overnight. Cells were washed three times in PBS and incubated for 1 h at room temperature with a fluorescent secondary antibody (Alexa 488 goat anti-rabbit, Invitrogen; 1:250 in PBS with 10% Thermo Blocker BSA #37525), washed three times with PBS, and mounted onto microscope slides with Prolong Gold anti-fade medium (Invitrogen) containing 4,6-diamidino-2-phenylindole stain as a marker for cell nuclei. Immunostaining experiments were performed in duplicate.

### RNA immunoprecipitation

RIP with control and DNMT antibodies was performed based on a previously published protocol^15^, with minor modifications. Day 1: primary neuronal cultures (∼250,000 neurons per culture well) were cross-linked with 1% formaldehyde in PBS, freshly supplemented with protease/phosphatase inhibitor cocktail (1/100th volume of Halt inhibitor cocktail (Pierce)), RNase inhibitor (40 U RNasin (Promega) per ml of buffer)) and 2 μM vanadyl complex. After incubation for 10 min at room temperature on a rocking platform, 2 M glycine was added to each well to a final concentration of 0.2 M to stop crosslinking, and rested on ice for 10 min. Cells were washed three times with ice-cold PBS (freshly supplemented with Halt cocktail, RNase inhibitor and vanadyl complex). Cells were lysed in RIP lysis buffer (50 mM HEPES, 10 mM NaCl, 1 mM EDTA, 0.5% NP-40, freshly supplemented with Halt cocktail, RNase inhibitor and vanadyl complex). Cells were detached with a cell scraper and placed on ice for 10 min to complete lysis. 1.2 × 10^7^ cells were collected and dounce homogenized (10 strokes with pestle A, 40 strokes with pestle B) and centrifuged for 10 min at 2,500*g* at 4 °C to pellet cell nuclei. After resuspension in RIP resuspension buffer (50 mM HEPES, 10 mM MgCl_2,_ freshly supplemented with 1X Halt cocktail, RNase inhibitor, and vanadyl complex), samples were sheared by sonication (three times for 5 s at 4 °C with BioRuptor Pico, with 30 s off time between pulses). DNase treatment was performed at 37 °C for 30 min, and EDTA was added to a concentration of 20 mM to stop the reaction. After adding RIP dilution buffer (50 mM HEPES, 150 mM NaCl, 1 mM EDTA, 0.5% NP-40, 0.1% Triton X-100, 0.1% sodium deoxycholate, freshly supplemented with Halt cocktail, RNase inhibitor and vanadyl complex), 100 μl of the sample was removed as the input control.

Immunoprecipitation for RIP was performed as follows. Samples were pre-cleared for 1 h with 25 μl fully re-suspended magnetic beads (Protein-A coated Dynabeads Beads, Invitrogen) at 4 °C on a rocking platform. Beads were removed in a magnetic field, and the remaining sample was divided equally for three IP reactions. Antibodies for IP reactions were: 5 μg IgG control (Rabbit IgG, Abcam ab46540), 5 μg DNMT1 antibody (Abcam ab87656) and 5 μg DNMT3a antibody (Abcam ab2850). Samples were incubated overnight on a rocking platform at 4 °C.

Day 2: 25 μl Protein-A coated Dynabeads were added and incubation was carried for 1 h at 4 °C on a rocking platform. Dynabeads were separated in a magnetic field and supernatant (unbound fraction) was removed. Immuno-RNA complexes were washed six times with RIP dilution buffer. After the final wash, beads were re-suspended in RIP reversal buffer (100 mM Tris-HCl, 200 mM NaCl, 1 mM EDTA, 1% SDS, freshly supplemented with RNase inhibitor and vanadyl complex) and 20 μl Proteinase K was added to each sample. Incubation was performed for 1 h at 42 °C, then 1 h for 65 °C. Supernatant (containing RNA) was removed and RNA was extracted with miRNeasy kit (Qiagen). DNase treatment was performed on column and RNA was eluted in 30 μl buffer EB (Qiagen). RT-qPCR was used to determine differences in RNA levels (compared with IgG control).

### RNA electrophoretic mobility shift assay (REMSA)

Mobility shift assays were conducted with custom synthetic Cy5.5-labelled 25-base RNA oligonucleotides (Sigma) and specified concentrations of full-length recombinant human DNMT1 protein (Sigma, SRP0126), recombinant human DNMT3a and DNMT3l protein (Sigma, SRP0396), or truncated murine DNMT3a protein containing only the catalytic methyltransferase domain (DNMT3a-CD, purified as previously described^49^). RNA oligonucleotides (1 nM) were incubated with DNMT protein (0–0.2 μM), IgG protein (0.2 μM), or 1 nM-100 nM poly-dI-dC (Sigma) in REMSA buffer (20 mM HEPES, 40 mM KCl, 1 mM EDTA, 0.2 mM DTT, 0.1 mg ml^−1^ BSA, 0.1% Tween-20, and 20% Glycerol) for 1 h at 37 °C, and REMSA was performed using native polyacrylamide gel electrophoresis (Bio-Rad Mini-Protean TGX Precast 4–20% gel). Electrophoretic mobility of Cy5.5-labelled RNA was assayed using fluorescence imaging on the Odyssey Infrared Imaging System (Li-Cor Biosciences). RNA-DNMT3a complex formation was quantified as the Cy5.5 signal intensity appearing at the higher band (corresponding to DNMT-bound RNA with lower electrophoretic mobility) divided by the total signal intensity (bound RNA plus free probe). Binding affinity (Kd) was calculated using non-linear regression (one-site-specific binding) in Prism 6.0 software (Graphpad), constraining Bmax equal to the theoretical maximum of 100% binding. Synthetic RNA probes showed no binding affinity for isomolar concentrations of proteins with similar mass (rabbit IgG, 150 kDa) or charge (BSA, pI=6.17) as full-length human DNMT3a (101 kDa, pI=6.57).

### *In vitro* methylation assay

*In vitro* methylation assays were performed with 10 ng of a 189-bp gDNA template from the *Fos* promoter (containing 15 CpG sites and amplified from *Fos* gDNA MeDIP primers). DNA was incubated for 1 h at 37 °C with 0.06 μM DNMT3a-3l (Sigma, SRP0396) and 0.5 mM S-(5′Adenosyl)-L-methyonine (SAM, Sigma) in REMSA buffer alongside DNA, protein and SAM only controls. For the methylation reaction with the added synthetic ecRNA, 5.16 μM ecRNA (ecRNA-1 oligonucleotide sequence) was added to account for the size differences between the RNA (25 nt, single-stranded) and DNA (189 bp, double-stranded) template and to provide ∼15x coverage of RNA for each DNA molecule. Purified DNA (0.2 ng) from each reaction was mixed with 500 ng sonicated rat genomic DNA to reduce non-specific binding and denatured at 95 °C for 10 min. Each reaction was diluted with 1x bind/wash buffer (0.5% NP-40, 1.1% TritonX100, 1.5 mM EDTA, 50 mM Tris-HCl, and 150 mM NaCl), and an input control was removed. To quantify DNA methylation, MeDIP was carried out with 10 μl magnetic beads (Protein-A coated Dynabeads Beads, Invitrogen) and 1 μl 5 mC antibody (Epigentek, #A-1014) for 1 h at 4 °C with rotation. Beads were washed 3 × with 1 × bind/wash buffer for 5 min/wash and re-suspended in buffer EB with 1% SDS. Proteinase K (Qiagen) was added and reactions were incubated at 50 °C for 1 h then 95 °C for 10 min to reverse binding. Beads were removed and IP reactions and inputs were purified using a PCR Purification Kit (Qiagen). Purified DNA was subjected to qPCR, and methylation levels were computed as a fraction of input DNA.

### RNA secondary structure prediction

Secondary structure of synthetic RNA oligonucleotides (Supplementary Fig. 5) was predicted using RNAfold software^50^ (via web server hosted at the University of Vienna), using minimum free energy and partition function algorithms.

### Anti-sense oligonucleotide (ASO) design and treatment

To manipulate *Fos* mRNA or ecRNA levels, we designed four 20 bp ASOs that targeted distinct transcripts from the *Fos* gene locus (Fig. 6a; Supplementary Data 3). ASOs targeting exon 3 of *Fos* mRNA or *Fos* 3′ ecRNA were synthesized with two chemical modifications: an all phosphorothioate backbone and five 2′ O-methyl RNA bases on each end of the oligonucleotide (Integrated DNA Technologies). These modifications have been shown to enhance ASO stability and improve affinity for the target RNA molecule^51^, and have successfully been employed for targeted manipulations in animal models of degenerative and developmental disorders^52^,^53^. Primary neuronal cultures were treated with vehicle (15 μl buffer EB, Qiagen) or ASO (15 μl in buffer EB, for a final concentration of 1.5 μM) and incubated for 72 h. Following ASO treatment, RNA was extracted (Qiagen RNeasy kit) and *Fos* mRNA and ecRNA levels were determined using RT-qPCR with custom primers (Supplementary Data 3). Gene expression was determined by normalizing to *Gapdh* (2^−ΔΔCt^).

### Methylated DNA immunoprecipitation

MeDIP was performed using a 5-methylcytosine antibody (4 μl per sample, mouse monoclonal, Epigentek #A-1014) as described previously^4^,^27^, with minor modifications. Genomic DNA was extracted (DNeasy Blood and Tissue Kit, Qiagen), treated with RNase A and quantified (Quant-IT HS dsDNA kit, Invitrogen) using the manufacturer's recommended protocols. 300 ng of DNA per sample was removed and sonicated (Bioruptor Pico, Diagenode) to 200- to 800-bp fragments for methylation analysis. Fragmented DNA was incubated for 1 h with 4 μl 5 mC antibody and methylated DNA was collected with protein-A−coated Dynabeads (Invitrogen), washed (1x wash buffer, MethylMiner kit, Invitrogen), extracted for 2 h at 60 °C with proteinase K in TE buffer with 1% SDS, and purified (Qiagen DNAeasy kit). To ensure adequate enrichment of methylated DNA fragments in each sample, we spiked samples with synthetic methylated and unmethylated control DNA fragments (1 pg each, Methyl Miner kit, Invitrogen) before MeDIP. Methylation at selected DNA regions was assayed via qPCR on a CFX96 real-time PCR system (Bio-Rad) with Sso Advanced chemistry. Ct values for IP samples were normalized to *Gapdh*, which did not change across samples. There were no differences in genomic DNA from input samples. There was no difference in methylated DNA enrichment between groups. The mean enrichment for synthetic methylated DNA spike-in controls over non-methylated fragments was >150-fold for each sample.

### Bisulfite sequencing

DNA methylation at the *Fos* gene promoter following *Fos* ecRNA ASO treatment was assayed using bisulfite sequencing. Genomic DNA was extracted (DNeasy Blood and Tissue Kit, Qiagen), treated with RNase A and quantified (Quant-IT HS dsDNA kit, Invitrogen) using the manufacturer's recommended protocols. 450 ng of DNA per sample underwent bisulfite conversion (EZ DNA Methylation Lightning kit, Zymo) and PCR amplification using bisulfite-compatible primer sets targeting the *Fos* promoter locus (Fig. 6e). PCR products were cloned using the TOPO-TA cloning system (Invitrogen) and sequenced using Sanger sequencing (UAB Heflin Genomics Core). 10–15 individual clones were sequenced per conversion, and the experiment was performed in quadruplicate (resulting in 41–45 clones/group).

### Western blotting

To determine whether ecRNA-mediated knockdown of *Fos* mRNA also resulted in a loss of Fos protein, we performed immunoblotting after 72 h incubation with *Fos* ecRNA ASOs. For each sample, 250,000 cells were washed with ice-cold Tris-buffered saline, lysed in 100 μl RIPA lysis buffer (50 mM Tris-HCl, 150 mM NaCl, 1% NP-40, 0.5% sodium deoxycholate, 0.1% SDS and 1X Halt protease and phosphatase inhibitor (Pierce)) for 30 min, and sonicated to reduce sample viscosity (Bioruptor Pico (Diagenode); 3 × 5 s, 30 s off time between pulses). Protein sample (20 μl) was boiled at 95 °C for 5 min with 4 × Laemmli buffer (Bio-Rad), separated on a 15% polyacrylamide gel, and transferred to a polyvinylidene difluoride membrane. Fos protein was detected with a rabbit polyclonal anti-Fos antibody (1:200; Santa Cruz #D2513), and imaged on an Odyssey Infrared Imaging System (Li-Cor Biosciences) using a goat anti-rabbit secondary (1:10,000; IR dye 800, Li-Cor Biosciences #827-08365). As a loading control, β-Tubulin was detected using a mouse anti-β-Tubulin antibody (1:1,000; Millipore #05-661) and imaged using a goat anti-mouse secondary antibody (1:10,000; IR dye 680, Li-Cor Biosciences #926–68,170). Protein levels were quantified in Odyssey Application Software v3.0 (Li-Cor Biosciences), and Fos intensity values were normalized to β-Tubulin for analysis.

### Animals

Male Sprague-Dawley rats, ∼90–120-day-old and weighing 250–350 g, were co-housed in plastic cages in an AAALAC-approved animal care facility on a 12-h light/dark cycle with food and water available *ad libitum*. All experiments were performed with naïve animals in the light phase of the light/dark cycleMBD-seq. All procedures were performed in accordance with the University of Alabama at Birmingham Institutional Animal Care and Use Committee. All animals were randomly assigned to respective groups.

### Stereotaxic surgery

Naïve adult Sprague-Dawley rats were anaesthetized with 4% isoflurane and secured in a stereotaxic apparatus (Kopf Instruments). During surgical procedures, an anaesthetic plane was maintained with 1–2.5% isoflurane. Under aseptic conditions, guide holes were drilled using stereotaxic coordinates (anteroposterior, −3.3 mm from bregma,±2.0 mm lateral from midline) to target the dorsal CA1 region of the hippocampus. All infusions were made using a gastight 30-gauge stainless steel injection needle (Hamilton Syringes) that extended into the infusion site (2.5 mm ventral to bregma). Bilateral microinfusions of 1 μl solution were made using a syringe pump (Harvard Apparatus) at a rate of 0.25 μl min^−1^. Injection needles remained in place for 5 min following infusion to allow for diffusion. Rats were infused bilaterally with either 1.5 μM of ASO in sterile saline or a vehicle control (saline alone) into the CA1 of the hippocampus. After infusions, guide holes were covered with sterile bone wax and surgical incision sites were closed with nylon sutures. Animals received buprenorphine for pain management.

### Contextual fear conditioning

To examine the role of *Fos* ecRNA in the adult brain in the context of learning and memory, we performed *in vivo* experiments with *Fos* ecRNA ASO-1, which demonstrated the most robust reduction of *Fos* ecRNA in neuronal cultures. *Fos* ecRNA ASO was delivered directly to the CA1 subregion of rat hippocampus via stereotaxic microinfusion. Knockdown was verified at 7 day following infusions with RT-qPCR on RNA extracted from dorsal CA1 tissue punches. Following recovery from surgery, animals were habituated to handling for 2 days before fear conditioning.

For both ASO and non-ASO experiments, contextual fear conditioning was conducted as previously described^2^,^3^. Animals were placed in a standard behavioural chamber (Med Associates) for a 7-min behavioural conditioning session. This session consisted of three electric shocks (1 s, 1 mA each) delivered to a metal floor grid every 2 min from the start of the behavioural session. After the final shock, animals remained in the chamber for 1 min. This training paradigm has previously been shown to induce robust and long-lasting contextual fear memory. To examine induction of *Fos* mRNA and ecRNA following fear conditioning, animals were killed at 1 h following behavioural training. Brains were rapidly removed and the CA1 of the hippocampus was dissected away from other hippocampal subregions for downstream analysis. To examine the effect of *Fos* ecRNA ASO treatment, we tested memory performance at 1 h (short-term memory) and 24 h (long-term memory) after the completion of training. For memory tests, animals were returned to the fear conditioning chamber and allowed to explore the environment for 7 min. During this time, we monitored freezing behaviour as a readout of fear-related memory, which was captured using high-speed video recording. Freezing behaviour was quantified as the time spent immobile, and was manually scored by three experimenters that were blind to treatment group.

### Open field test

A standard open field arena (43 × 43 cm; Med Associates) was used to assess locomotor activity and anxiety-like behaviour. Seven days following vehicle or *Fos* ecRNA ASO infusions into the CA1 of the hippocampus, rats were placed in the open field arena for a 30-min period. Activity was tracked using automated video tracking software (CinePlex Studio, Plexon Inc.). Distance travelled (in cm) was used to quantify total locomotor activity, and time spent in the center of the open field (defined as 18 cm square in the middle of the field) was used to quantify anxiety-like behaviour.

### Statistical analyses

Transcriptional and epigenetic differences from PCR experiments were compared with one- or two-way ANOVA with Tukey or Sidak *post hoc* tests, or *t*-tests where appropriate. Bisulfite sequencing data was analysed using a *χ*^2^ test with Yates' correction to compare the difference in methylated CpGs between vehicle and ASO treatment, or with a two-way ANOVA followed by Sidak's *post hoc* test (with correction for multiple comparisons at individual CpG sites). Genome-wide correlations between ecRNA quartile and mRNA or promoter DNA methylation were performed with a one-way ANOVA followed by a *post hoc* test for a linear trend between column mean and left-to-right column order. Statistical significance was designated at *α*=0.05 for all analyses. Statistical and graphical analyses were performed with Graphpad software (Prism). Where necessary, statistical assumptions (for example, normality for parametric tests) were formally tested. Graphical analyses were performed in Matlab (v. R2014b) and DeepTools^54^ (via Galaxy server).

### Data availability

Sequencing data that support the findings of this study have been deposited in Gene Expression Omnibus (GEO) with the accession number GSE64988 (http://www.ncbi.nlm.nih.gov/geo/query/acc.cgi?acc=GSE64988). All relevant data that support the findings of this study are available on request from the corresponding author (J.J.D.).

## Additional information

**How to cite this article:** Savell, K. E. *et al*. Extra-coding RNAs regulate neuronal DNA methylation dynamics. *Nat. Commun.* 7:12091 doi: 10.1038/ncomms12091 (2016).

## Supplementary Material

Supplementary Information Supplementary Figures 1-7

Supplementary Data 1 Genes with altered expression following KCl-induced neuronal depolarization

Supplementary Data 2 Gene clusters identified by hierarchical clustering analysis

Supplementary Data 3 PCR primer and ASO sequences
