# Supplementary material for: Extra-coding RNAs regulate neuronal DNA methylation dynamics
Source: Nat Commun. 2016 Jul 7;7:12091. doi: 10.1038/ncomms12091 (PMC4941050; doi:10.1038/ncomms12091)
Supplement: Supplementary Information — Supplementary Figures 1-7 [file ncomms12091-s1.pdf]

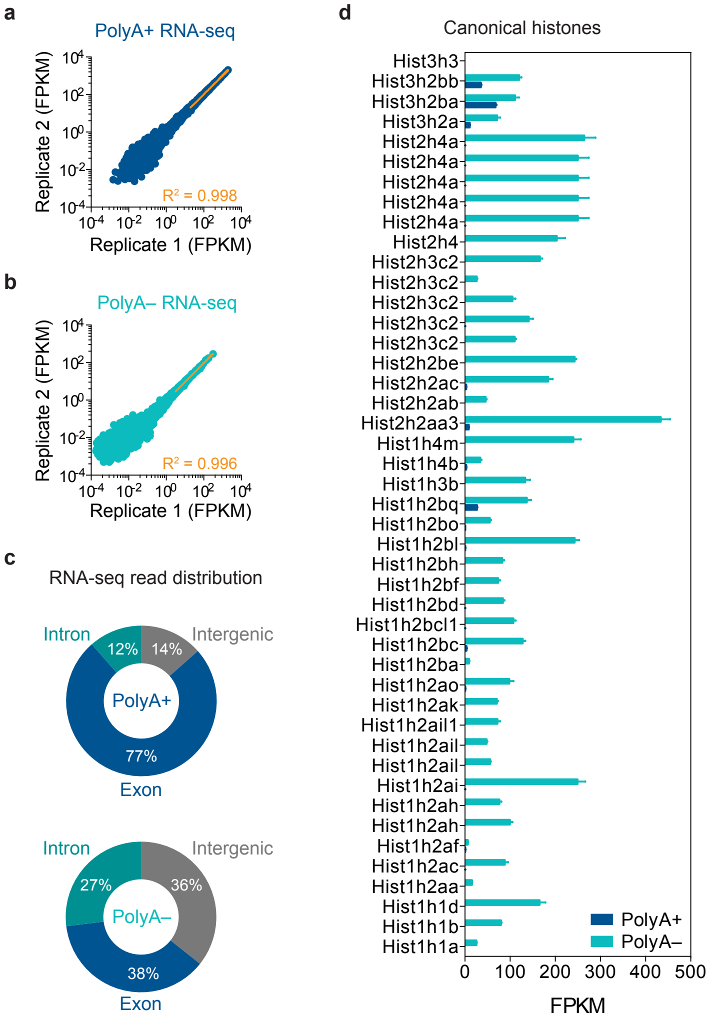

Supplementary Figure 1. PolyA+ and PolyA– RNA-seq validation. **a,** Correlation between individual biological replicates for PolyA+ RNA-seq (linear regression, $P < 0.0001$). **b,** Correlation between individual biological replicates for PolyA– RNA-seq (linear regression, $P < 0.0001$). **c,** Distribution of mapped sequenced reads in PolyA+ (top) and PolyA– (bottom) RNA-seq. Percentages sum to greater than 100 due to read overhang between features. **d,** Expression estimates from PolyA+ and PolyA– RNA-seq for canonical histone transcripts, which are known to lack polyadenylation signals. Reads mapping to these transcripts were more abundant in the PolyA– library for all histone genes, indicating specific selection of PolyA– transcripts in the PolyA– RNA-seq. Data are expressed as mean ± s.e.m.

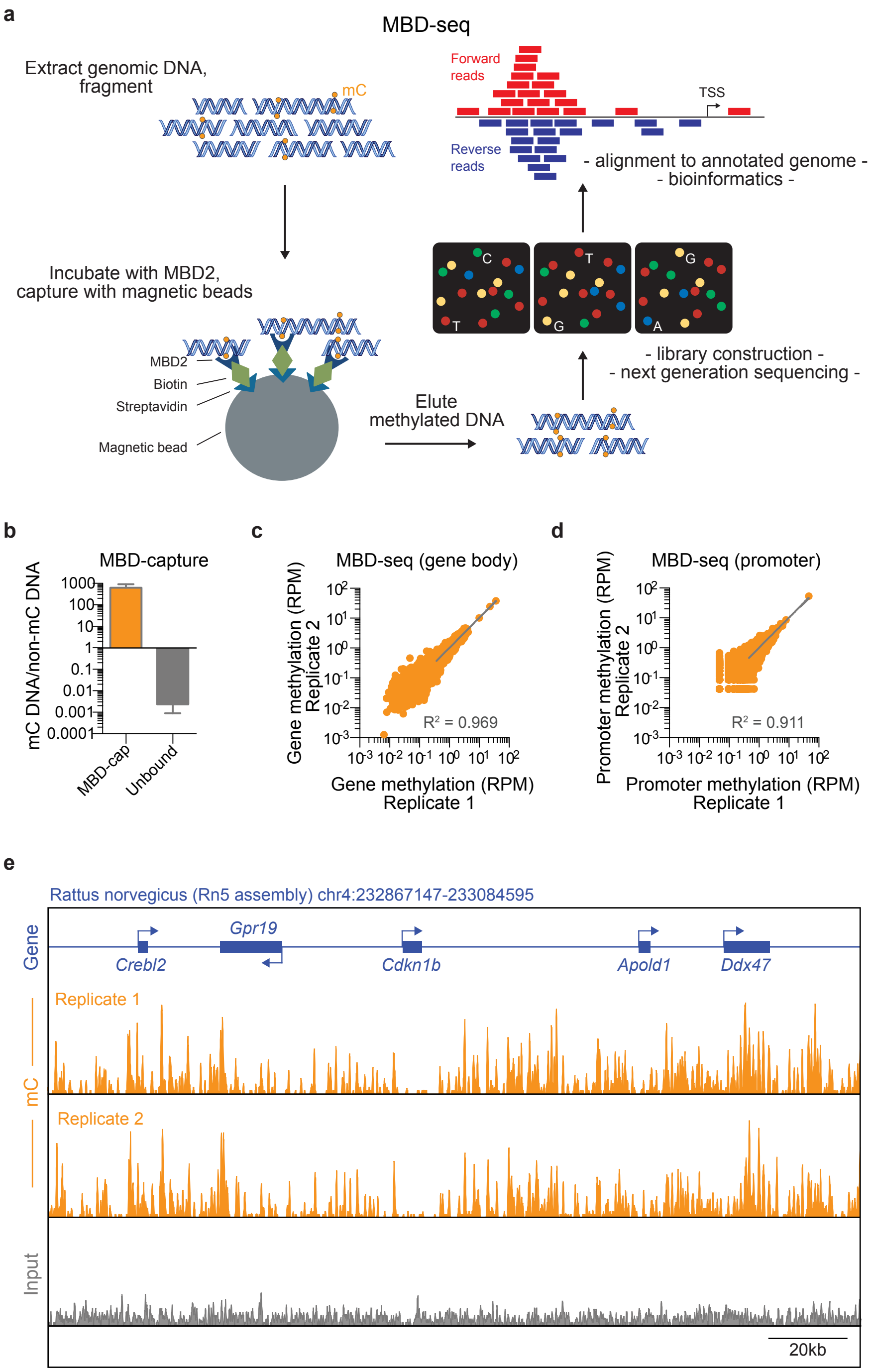

Supplementary Figure 2. MBD-seq pipeline and validation. **a,** MBD-seq workflow. **b,** Validation of methylated DNA capture with MBD-IP. Incubation with MBD2 resulted in robust enrichment of methylated DNA in IP sample and depletion from unbound fraction (n = 4 per group; 2-tailed Mann-Whitney test; P = 0.0286). Data are expressed as mean ± s.e.m. **c,** Correlation of gene body methylation levels between individual biological replicates in MBD-seq (linear regression, P < 0.0001 for each comparison). **d,** Correlation of promoter methylation levels between individual biological replicates in MBD-seq. **e,** Representative genomic locus highlights DNA methylation patterns surrounding gene bodies and lack of defined peaks in input (non-IP) sample.

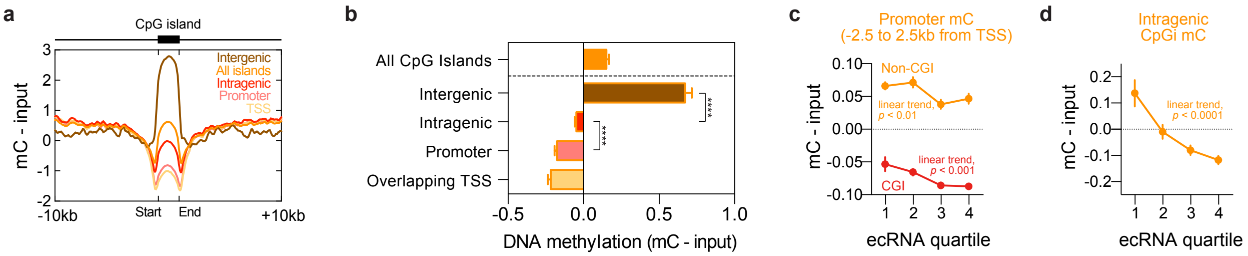

Supplementary Figure 3. Genome-wide DNA methylation by CpG island status. **a,** Meta-feature profile of DNA methylation at CpG islands, stratified by genomic location. TSS-spanning CpG islands are hypomethylated, whereas intergenic CpG islands are hypermethylated. Data represents average methylation across categories, aligned to CpG island start and end locations. **b,** Comparison of CpG island methylation by genomic feature (n = 2683-11302 per group; one-way ANOVA, $F(4,35696) = 176.2$; $P < 0.0001$, Tukey's post-hoc test for individual comparisons). **c,** Promoter DNA methylation according to ecRNA rank, stratified by presence (red points) or absence (orange points) of CpG island (. **d,** DNA methylation at intragenic CpG islands, by ecRNA rank of corresponding gene. ecRNA levels are significantly correlated with methylation of intragenic CpG islands.

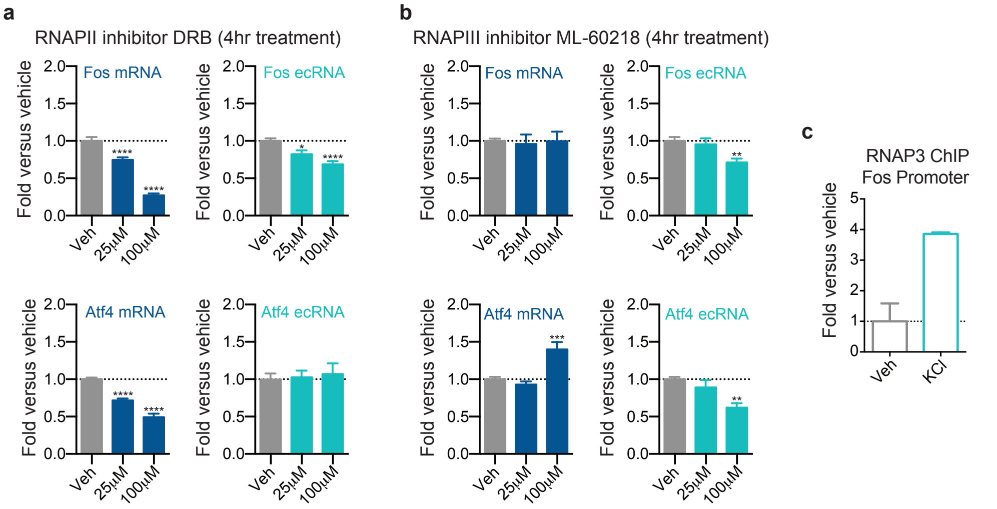

Supplementary Figure 4. *Fos* and *Atf4* ecRNA are differentially regulated by RNA polymerases. **a,** Treatment with the RNA polymerase II-dependent transcriptional inhibitor 5,6-dichloro-1-beta-D-ribofuranosylbenzimidazole (DRB) blocked *Fos* mRNA and ecRNA production. DRB treatment had no effect on *Atf4* ecRNA but decreased *Atf4* mRNA in a dose-dependent manner. **b,** Treatment with the RNA polymerase III inhibitor ML-60218 had no effect on *Fos* or *Atf4* mRNA but decreased *Fos* and *Atf4* ecRNA (n = 17-18 per group for DRB experiments and 15-16 per group for ML-60218 experiments; one-way ANOVA, Tukey's post-hoc test for multiple comparisons). **c**, After KCl induction, RNA polymerase III binding increases at the *Fos* promoter (n = 2 per group; Student's t-test versus vehicle, $P =$ 0.0396). All data are expressed as mean ± s.e.m. Individual comparisons, *P < 0.05, **P < 0.01, ***P < 0.001, and ****P <0.0001.

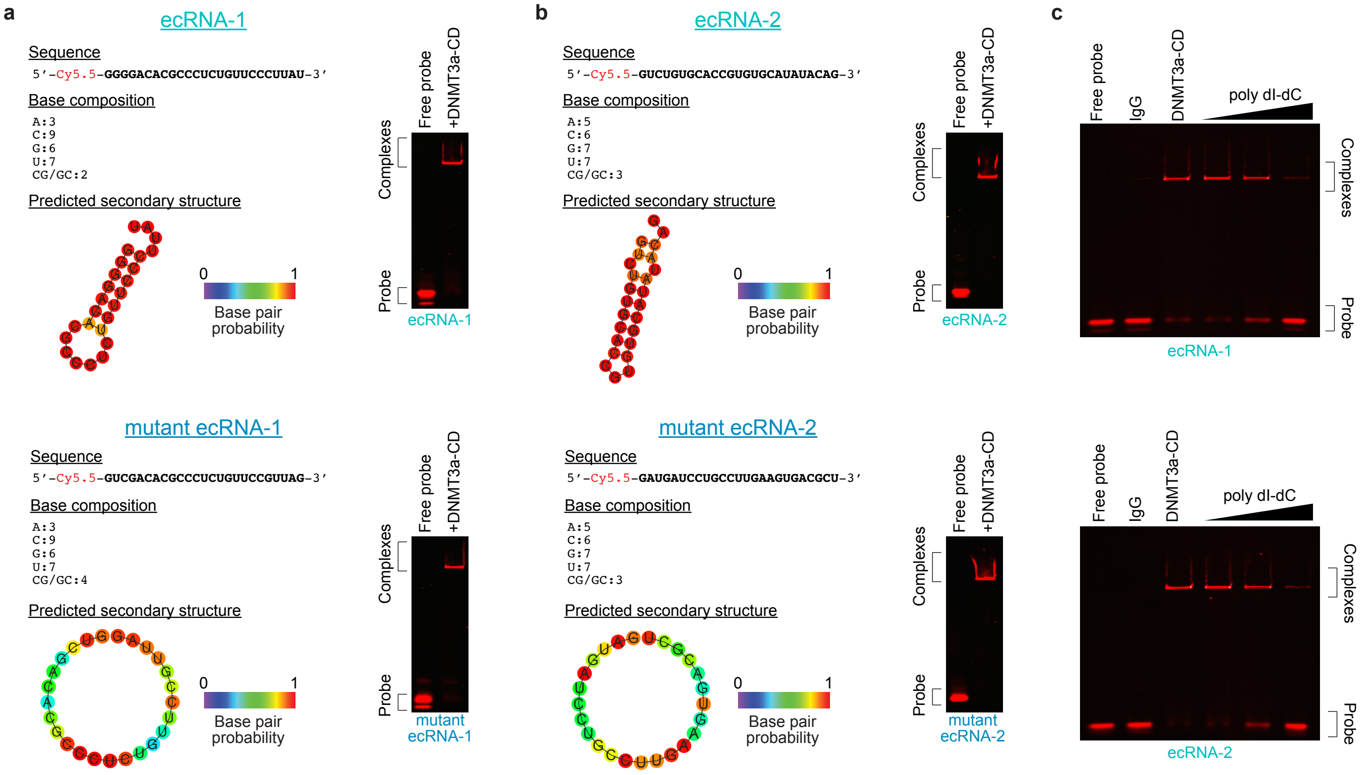

Supplementary Figure 5. ecRNA interactions with DNMT3a are not regulated by stem-loop secondary structure and are maintained in the presence of a non-specific competitor. **a,** Base sequence, composition, and predicted secondary structure for synthetic ecRNA-1 and mutant ecRNA-1 probes. Mutant ecRNA-1 possesses identical base composition but lacks secondary structure. DNMT3a-CD binding was not impaired by removal of secondary structure. **b,** Base sequence, composition, and predicted secondary structure for synthetic ecRNA-2 and mutant ecRNA-2 probes. Mutant ecRNA-2 possesses identical base composition but lacks secondary structure. DNMT3a-CD binding is not altered in mutant ecRNA-2. **c,** ecRNA-1 and ecRNA-2 probes (1nM) do not bind to IgG (0.2μM). Increasing amounts of the non-specific competitor poly dI-dC (1nM-100nM) does not abolish Fos ecRNA binding to DNMT3a-CD.

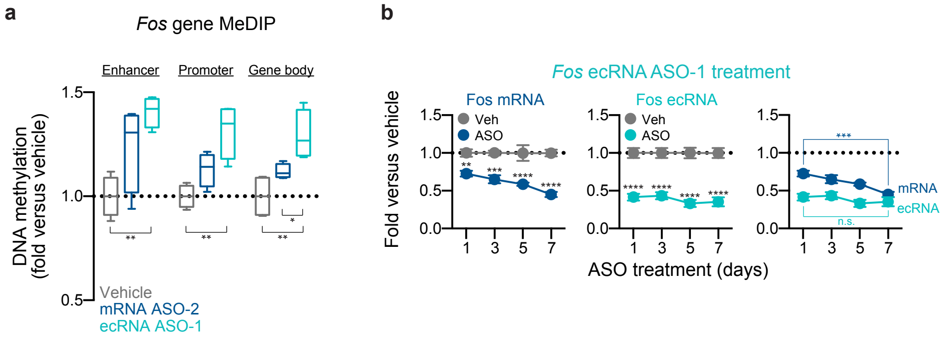

Supplementary Figure 6. ASO mediated knockdown of *Fos* mRNA and ecRNA in cortical cultures. **a,** *Fos* ecRNA knockdown resulted in increased enhancer, promoter, and gene body methylation (n = 4 per group; two-way ANOVA versus vehicle, Tukey's post-hoc test for multiple comparisons). **b**, ASO mediated ecRNA knockdown persisted for up to 7 days, and resulted in a progressive decline in *Fos* mRNA expression (n = 6 per group, two-way ANOVA, Sidak's post-hoc test for multiple comparisons). Data are expressed as mean ± s.e.m. Individual comparisons, *$P < 0.05$, **$P < 0.01$, ***$P < 0.001$, and ****$P < 0.0001$.

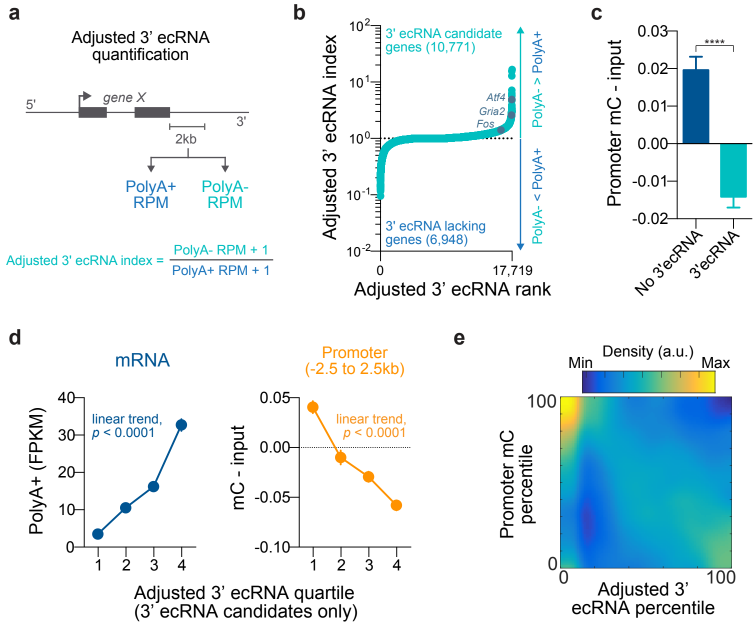

Supplementary Figure 7. Alternative analysis confirms genome-wide relationship between ecRNA and promoter DNA methylation. **a,** Alternative ecRNA quantification adjusts ecRNA levels for PolyA+ expression for the same locus, removing potential contamination from overlapping genes or incorrect gene annotations. **b,** Alternative analysis revealed at least 10,771 genes with 3' PolyA- RPM greater than 3' PolyA+ RPM. **c,** This group of genes exhibited less promoter methylation than genes lacking 3'ecRNA production. **d-e,** 3' ecRNA status tracks mRNA and DNA methylation.
